# A Meta-Analysis of Magnetic Resonance Spectroscopy Studies on Glutamatergic Neurometabolite Levels in Major Depressive Disorder

**DOI:** 10.1155/da/5180077

**Published:** 2025-07-31

**Authors:** Shiwan Tao, Renhao Deng, Menghan Wei, Yunqi Huang, Huan Sun, Shuhan Yang, Shen Li, Chutian Xiao, Mingli Li

**Affiliations:** ^1^Mental Health Center, West China Hospital of Sichuan University, Chengdu, Sichuan, China; ^2^Mental Health Center and Psychiatric Laboratory, West China Hospital of Sichuan University, Chengdu, Sichuan, China; ^3^Department of Psychiatry, West China Second University Hospital, Sichuan University, Chengdu, China; ^4^Department of Urology, The Sixth Affiliated Hospital, Sun Yat-Sen University, Guangzhou, China

**Keywords:** glutamatergic neurometabolites, major depressive disorder, meta-analysis

## Abstract

Glutamatergic neurometabolite dysregulation has recently garnered attention in the pathophysiology of major depressive disorder (MDD). However, studies have reported heterogeneous results for changes in neurometabolite levels across brain regions and whether these changes are related to antidepressant intervention. Herein, we performed a meta-analysis to investigate consistent findings by searching PubMed, Embase, MEDLINE, PsycINFO, and SinoMed from the start date of these databases to May 2023 (PROSPERO#CRD42023405205). Among the 2529 publications screened, 55 studies were included in the meta-analysis, with 1400 MDD patients and 1322 healthy controls. The results revealed significant decreases in Glx (glutamate + glutamine) in the anterior cingulate cortex (ACC) and prefrontal cortex (PFC), glutamate in the ACC and γ-aminobutyric acid in the occipital cortex in MDD patients compared with healthy controls. However, changes in neurometabolite levels from pre to posttherapeutic-intervention in MDD patients were not significant. Heterogeneity was moderate-to-high across all neurotransmitters and brain regions. Nonetheless, these findings inform current translation efforts for MDD research.

## 1. Introduction

Major depressive disorder (MDD) is a prevalent mental disorder worldwide, affecting 5.3% of the global population [1]. It ranks as the 13^th^ leading cause of disability-adjusted life-years (DALYs) and accounted for the largest proportion of mental disorder-related DALYs [2] at 37.3% [3] in 2019. MDD is diagnosed on the basis of criteria in the Diagnostic and Statistical Manual of Mental Disorders (DSM) [4] or the International Classification of Disease (ICD) [5]. Both systems diagnose patients based on clinical symptoms, illness course, and functional impairment. However, depressive symptoms in the general population are highly heterogeneous, continuous in severity, and easily confused with subthreshold depressive symptoms in clinical practice. [6] Identifying reliable and objective biomarkers to aid in the early diagnosis and treatment of MDD is important. Previous studies have reported that the duration of untreated depression significantly affectes clinical outcomes, with shorter duration linked to more favorable outcomes and reduced disability, [7, 8] whereas longer episodes before treatment predict recurrence. [9] Early diagnosis may help shorten this duration, alleviating patient suffering.

The most widely used and effective therapeutic strategy for MDD is the use of antidepressants, [10] especially second-generation antidepressants such as selective serotonin reuptake inhibitors (SSRIs) and serotonin and norepinephrine reuptake inhibitors (SNRIs). However, the prognosis of patients with MDD is highly heterogeneous, around with approximately 30% of patients developing treatment-resistant depression (TRD) [11]. This limitation has led researchers to identify novel therapeutic targets beyond monoamine systems, and the glutamatergic system in the central nervous system (CNS) is promising. Studies have shown that traditional monoaminergic-based antidepressants can regulate glutamate (Glu) release and transmission, and reduce the function of Glu receptors in animal models [12]. Moreover, the recent approval of antidepressant (es)ketamine, [13] an antagonist of N-methyl-D-aspartate (NMDA) receptors, a type of ionotropic Glu receptor (iGluR), has further emphasized the role of glutamatergic system modulation in depression [14, 15].

As the most common brain excitatory and inhibitory neurotransmitters, both Glu and gamma-aminobutyric acid (GABA), or their balance in different areas of the brain, are considered key pathogenic pathways in depression and potential targets of antidepressant treatments [14, 16]. Reduced levels of Glu and/or GABA as well as an imbalance in the Glu/GABA ratio have previously been reported to be associated with depression, reflecting impaired excitatory/inhibitory (E/I) neurotransmission in MDD [17–19], Although the mechanisms are incompletely understood, E/I imbalance may contribute to depression by dysregulating receptor expression, altering neuronal morphology, and impairing functional patterns within specific brain regions or large brain networks. For example, Glu mediates most excitatory neurotransmission in mammals through metabotropic Glu receptors (mGluRs) and iGluRs [20]. The expression level of mGluR5 in the medial prefrontal cortex (mPFC) and ventral hippocampus neurons mediates chronic stress and depression-like behaviors in mice models [21], while mGluR5/mGluR7 antagonists have shown antidepressant potential in preclinical and clinical studies [22, 23]. Reduced GABA concentrations in the occipital cortex (OCC) of MDD patients [24] and in plasma, cerebrospinal fluid, and cortical tissue may be the most compelling evidence of GABAergic deficits associated with MDD [19, 25–29]. Moreover, postmortem studies have shown that the size and density of GABAergic neurons are significantly reduced in the prefrontal [30, 31] and OCC layers [32] of MDD patients. In preclinical studies, antidepressants and electroconvulsive therapy were suggested to alter or reverse the decrease in GABA in rat animal models [33, 34].

Most of the aforementioned studies were conducted in animal models. Previous research has yielded inconsistent findings regarding the concentrations of glutamatergic neurotransmitters in the brains of MDD patients compared to healthy controls, therefore, further systematic reviews are warranted. While traditional monoaminergic-based antidepressants can modulate glutamatergic neurotransmitter levels, their effects on concentration trends remain inconsistent, highlighting the need for further investigation. Magnetic resonance spectroscopy (MRS) is an excellent noninvasive imaging technique used to measure changes in endogenous metabolites in the human brain in vivo. It uses signals from hydrogen protons to determine concentrations of neurometabolites such as GABA, Glu, glutamine (Gln), creatine (Cr) and lactate [35]. MRS is an important approach in studies investigating potential biomarkers for the precise diagnosis and treatment response of MDD patients. A previous meta-analysis revealed a decreased level of Gln + Glu (Glx) within the mPFC in patients with depression compared with controls [36]. However, this meta-analysis did not include studies measuring GABA neurometabolites, and differences in Glu neurotransmitter levels pre and post-therapeutic-intervention were not compared. Additionally, articles published after 2019 can provide more research progress and discoveries.

In the present study, we conducted a meta-analysis study to compare the levels of neurometabolites (Gln, Glu, Glx and GABA) in the prefrontal cortex (PFC), anterior cingulate cortex (ACC), OCC and hippocampus between MDD patients and healthy controls. We also compared the neurometabolite levels of MDD patients at baseline and follow-up. To increase the homogeneity of the included samples, we applied strict inclusion and exclusion criteria, excluding patients with comorbidities other than anxiety disorders [37–39]. This study aimed to identify relatively stable differences in neurotransmitter levels between MDD patients and controls, and to determine whether these changes in neurotransmitter levels are associated with depressive severity. This study is beneficial because it highlights the possibility of using noninvasive measurement of neurotransmitter concentrations via MRS as potentially reliable biomarkers for clinical diagnosis and treatment.

## 2. Methods

### 2.1. Protocol Registration

We followed the Transparent Reporting of Systematic Reviews and Meta-Analyses (PRISMA) statement (http://www.prisma-statement.org/) and uploaded the full protocol to the website of International Prospective Register of Systematic Reviews (https://www.crd.york.ac.uk/PROSPERO/), #CRD42023405205.

### 2.2. Search Strategies

The search was performed in PubMed, Embase, MEDLINE, PsycINFO, and SinoMed from the start date of these databases to May 2023. We used the following search terms: depress*⁣*^*∗*^ AND (glutam*⁣*^*∗*^ OR Glx OR GABA OR “*γ*-aminobutyric acid” OR “Gamma-aminobutyric acid") AND (MRS OR “magnetic resonance spectroscopy").

### 2.3. Inclusion Criteria and Exclusion Criteria

The inclusion criteria were as follows: (1) Patients who met the DSM 3rd, 4th, or 5th edition criteria for MDD or met the ICD-10 or ICD-11 for MDD; (2) patients whose Gln, Glu, Glx, or GABA levels in the brain were compared via MRS between patients with MDD and healthy controls, or between MDD patients pre and postintervention; (3) studies included at least five subjects in each group; (4) the mean differences between the two groups were presented.

The exclusion criteria or the analyses comparing neurometabolite levels between participants with and without MDD were as follows: (1) The review and conference abstracts; (2) the subjects were nonhuman; (3) the study did not include healthy controls; (4) the study was not written in English or in Chinese; (5) the MDD patients comorbidities with other mental disorders except anxiety disorder, because researches presented the high comorbidity rate of the MDD patients and anxiety disorder (higher than 45%) [37–39]. The details of the comorbid anxiety disorders in the MDD patients included in each study are provided in Table S1.

The exclusion criteria for comparison between MDD patients' baseline and follow-up neurometabolite levels were as follows: (1) Review and conference abstracts; (2) nonhuman; (3) did not report patients' neurometabolite levels or reported only patients' neurometabolite levels at one point; (4) did not write in English or in Chinese; (5) had comorbidities with other mental disorders except anxiety disorder [37–39].

### 2.4. Study Selection and Data Extraction

The study selection and data extraction were independently completed by two authors and cross-checked. In cases of discrepancies, a third author was consulted to reach a consensus. EndNote was used to select studies, and Excel was used to record the extracted data. If different studies reported data from the overlapping sample, the study with the larger sample size was included. The data we extracted contain general information (sample size, age, sex, and publication year), clinical information (therapy strategies, the scores of scales that are related to the situation of MDD), MRS parameters and the concentrations of GABA, Glu, Gln, and Glx (mean and standard deviation). Each metabolite was included in the meta-analysis only if at least four studies investigated it in the same brain region.

### 2.5. The Outcomes

The primary outcomes of the comparisons between the MDD patients and healthy controls were the differences in the GABA, Glu, Gln, and Glx levels between the patients and the controls in the brain regions of interests (ROIs), including the PFC, ACC, OCC, and hippocampus.

The primary outcomes of the comparisons between MDD patients at baseline and follow-up were the differences in the GABA, Glu, Gln, and Glx levels in the ROI brain regions, including the PFC, ACC, OCC, and hippocampus.

The ACC included both the mPFC and ACC since their ROIs often spatially overlap. The PFC contains the mPFC, dorsolateral prefrontal cortex (dlPFC), ventromedial prefrontal cortex (vmPFC) and inferior frontal cortex (IFG). If studies reported two laterals ROIs, we selected the lateral region which was reported in most studies.

### 2.6. Data Analysis

All analyses were performed using R Statistical Software (v4.2.1; [40]). The standardized mean difference (SMD) method was used to generate effect sizes, and the random-effects model was used to pool the effect sizes in the meta-analysis. For the case-control studies, we defined the patients as the experimental group, and for the longitudinal studies, we defined the baseline as the experimental group. We then used *Z*-statistics to test the pooled effect. Pooled effects with *p*-value less than 0.05 were considered significant. The between-study heterogeneity in the meta-analysis was assessed using the *Q*-test and *I*^2^ index. For those *I*^2^ indices higher than 50%, which were considered significantly heterogeneous, we further detected outlier studies and performed meta-analyses without them to validate the robustness of our results (if a study's confidence interval [CI] did not overlap with the CI of the pooled effect, we regarded the study as an outlier). We also performed subgroup analysis on the MRS reference and treatment data to identify the source of heterogeneity in the comparisons between cases and controls. *Q*-statistics was used to test for subgroup differences. If the *p*-value for the *Q*-test was less than 0.05 and the study size for each subgroup was greater than three, the subgroup analyses were considered significant (i.e., the pooled effect size was different among subgroups). Considering the small sample size, we skipped the subgroup analyses for comparisons between MDD patients' baseline and follow-up neurometabolite levels.

Meta-regression was conducted to predict the pooled effect size with the patients' sex, age, and study sample size. The coefficient with 95% CI represented the change in effect size for a one-unit change in predictor (i.e., patients' sex, age, and sample size of a study). *Q*_M_ statistics were used to test the regression model and *Z*-statistics were used to test the coefficient of each predictor. A *p*-value less than 0.05 was considered significant.

Funnel plots and Egger regression tests [41] were performed to detect publication bias when the involving study number was more than 10 [42], otherwise the Doi plot and Luis Furuya-Kanamori (LFK) index [43] was performed. For region-specific neurometabolites with high between-study heterogeneity (i.e., *I*^2^≈75%), we did not conduct publication bias analysis according to Aert's suggestion [44]. We did not conduct the subgroup analyses for comparison between MDD patients' baseline and follow-up neurometabolite levels due to the limited sample size.

We utilized the R package “meta” (v7.0.0; [45]) to perform the effect size calculation, data synthesis, heterogeneity evaluation, forest plot generation, the subgroup analysis, the meta-regression, and the publication bias detection. We utilized the R package “metasens” (v1.5.2; [46]) to generate the Doi plot and the LFK index.

## 3. Results

**Figure 1:**
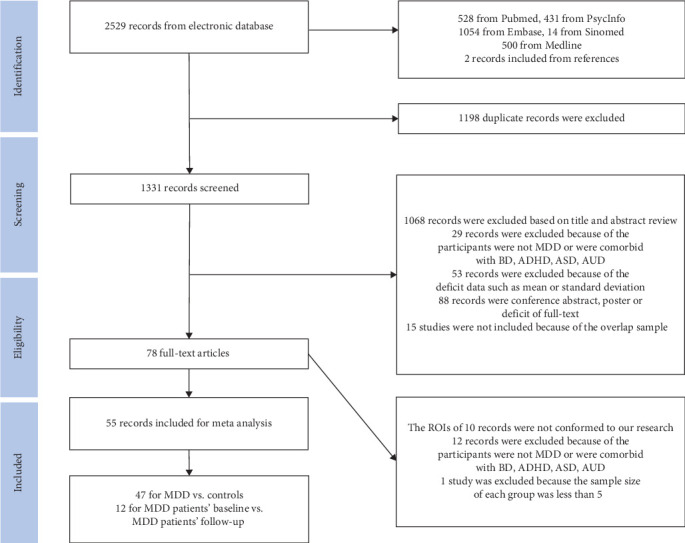
The procedure of study selection. The pipeline has been registered according to preferred reporting items for systematic reviews and meta-analysis (PRISMA). We finally included 55 studies for further meta-analysis. ROI, the brain regions of interest. AUD, alcohol use disorder; ADHD, attention-deficit/hyperactivity disorder; ASD, autism spectrum disorder; BD, bipolar disorder; MDD, major depressive disorder.

Finally, 47 studies comparing MDD patients with controls were included [17, 18, 24, 47–90] (Table 1). Twelve studies concerning therapeutic interventions were included [17, 70, 77, 88, 91–98] (Table S2). The study selection procedure is shown in Figure 1.

The results of the number of participants and *I*^2^ were as follows (Tables 2 and 3). We found that Glx in the ACC and PFC, Glu in the ACC and GABA in the OCC were significantly decreased in MDD patients compared with controls. However, other results did not reveal significant differences between patients with MDD and healthy controls (Figure S1), and there were no significant differences between patients pre and postintervention (Figure S2).

### 3.1. Glx in the ACC Region is Lower in MDD Patients Than in Healthy Controls

**Figure 2:**
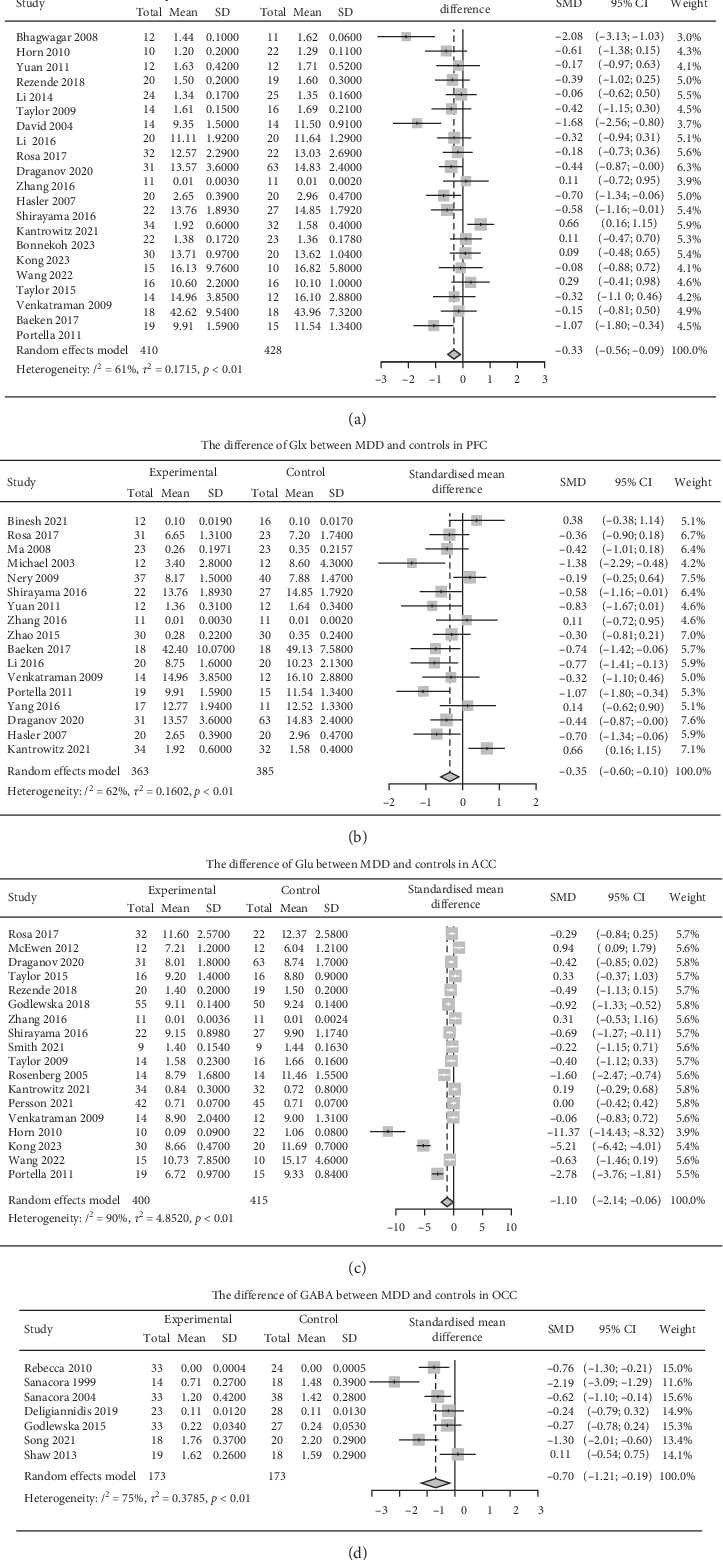
Study effect sizes of neurometabolites in brain regions that represent significant differences between MDD and controls. Forest plots show the pooled effect sizes (i.e., standardized mean difference with 95% CI) of random-effects meta-analysis on differences in various brain neurometabolite ((A) Glx in ACC; (B) Glx in PFC; (C) Glu in ACC; (D) GABA in OCC) levels between MDD patients and controls. The diamond markers display the pooled effect size of our random effects model. Rows of the forest plot list studies included in the meta-analysis. For each study, the forest plots lay out its author's name, publication year, study size, effect size, and study weight. ACC, anterior cingulate cortex; CIs, confidence intervals; GABA, γ-aminobutyric acid; Glu, glutamate; OCC, occipital cortex; PFC, prefrontal cortex; SMD, standardized mean difference. Glx, glutamine + glutamate; *I*^2^, an index of heterogeneity; *p*, for describing the significance of heterogeneity.

The pooled analysis revealed a significant reduction in Glx levels in the ACC among MDD patients compared with controls (SMD, 95% CI: −0.33, −0.56 to −0.09) (Figure 2A), with notable heterogeneity (*I*^2^ = 61.1%). After excluding outliers [18, 47, 83], the heterogeneity decreaed to 0.0%, and the difference between patients and controls remained significant (SMD, 95% CI: −0.27, −0.42 to −0.12) (Table S3). Subgroup analysis showed no significant differences between the treated and untreated subgroups, or between the subgroups when Cr or water was used as reference (*p*  > 0.05 in Tables S4 and S5). Meta-regression analysis indicated that patient sample size (coefficient = 0.03, *Q*_M_ = 4.41, *p*=0.04) was associated with the pooled effect size of Glx in the ACC (Table S6). To assess potential publication bias, a funnel plot (Figure S3) and Egger regression test (Table S7) were conducted for Glx in the ACC (21 studies), revealing significant publication bias (*p*=0.027).

### 3.2. Glx in the PFC Region is Lower in MDD Patients Than in Healthy Controls

Glx levels in the PFC were significantly lower in MDD patients compared to controls (SMD, 95% CI: −0.35, −0.60 to −0.10) (Figure 2B), with substantial heterogeneity (*I*^2^ = 62.2%). After excluding an outlier [18], the difference remained significant (SMD, 95% CI: −0.42, −0.62 to −0.21), and heterogeneity decreased to 43.9% (Table S3). Subgroup analysis and meta-regression did not reveal significant findings (*p*  > 0.05 in Tables S4–S6). Additionally, no significant publication bias was observed in the funnel plot (Figure S3) or Egger regression test (Table S7; *p* > 0.05).

### 3.3. Glu in the ACC Region is Lower in MDD Patients Than in Healthy Controls

Glu levels in the ACC were significantly lower in MDD patients compared to controls (SMD, 95% CI: −1.10, −2.14 to −0.06) (Figure 2C), but there was very high heterogeneity (*I*^2^ = 90.3%). After removing outliers [52, 59, 85], the *I*^2^ decreased to 74.8%, and the pooled effect remained significant (SMD, 95% CI: −0.47, −0.83 to −0.12) (Table S3). Subgroup analysis and meta-regression analysis did not show significant results (*p*  > 0.05 in Tables S4–S6). Owing to the high heterogeneity, we did not perform publication bias analyses for Glu in the ACC (*I*^2^ = 90%) (Section 2).

### 3.4. GABA in the OCC Region is Lower in MDD Patients Than in Healthy Controls

Another notable difference was observed in GABA levels in the OCC. Patients with MDD had lower GABA levels than controls did (SMD, 95% CI: −0.70, −1.21 to −0.19) (Figure 2D), with substantial heterogeneity (*I*^2^ = 74.9%). After excluding outliers [24], the heterogeneity decreased to 54.9%, and the difference between patients and controls remained significant (SMD, 95% CI: −0.50, −0.84 to −0.15) (Table S3). Subgroup analysis and meta-regression analysis did not yield significant findings (*p*  > 0.05 in Tables S4–S6). Given the high heterogeneity, publication bias analyses were not conducted for GABA in OCC (*I*^2^ = 75%) (Section 2).

### 3.5. Comparison Between Pre and Postintervention Patients

No significant differences were dectected between pre and postintervention measurements for any metabolite (Glx, Glu, and GABA in the ACC and PFC) in regions with at least four studies (Figure S2). Heterogeneity in these comparisons was low across all regions except for GABA in the PFC (*I*^2^ = 50.2%), although sensitivity analysis detected no outliers (Table S8). Subgroup analyses were not performed for baseline vs. follow-up comparisons as prespecified. Meta-regression analysis of the intervention comparisons revealed no significant associations (Table S9).

The Cochrane Handbook advises caution in interpreting meta-regression results when fewer than 10 studies are included. Given the limited study numbers for most region-specific metabolites in intervention comparisons, these findings require cautious interpretation. Doi plot analyses and LFK index calculations were also performed (Figure S4 and S5).

## 4. Discussion

Our meta-analysis highlights significant regional differences in the levels of glutamatergic metabolites including Glu, Gln, Glx, and GABA, in the brain between MDD patients and controls. Specifically, we found significantly decreased Glx in ACC and PFC, as well as a reduction in Glu in the ACC and GABA in the OCC. After removing outliers, the GABA level in the ACC became significantly lower in patients than in healthy controls. However, no significant changes were detected between baseline and the intervention in the MDD patients.

Our analysis revealed decreased Glx levels in the ACC of MDD patients compared with healthy controls. Given that the ROI of the ACC in our study included both the mPFC and ACC (Section 2), we considered this result consistent with a published meta-analysis [36], which revealed that Glx levels were decreased in the mPFC in MDD patients. Nevertheless, this earlier meta-analysis did not find significant differences in Glu levels between the MDD patients and healthy controls. In contrast, our study revealed significantly reduced Glu levels in the ACC region. This discrepancy may be partially explained by the presence of MDD comorbidities. The earlier meta-analysis did not exclude MDD patients comorbid with ASD, ADHD, and others, whereas our study applied more stringent inclusion criteria, excluding studies involving MDD comorbidities except for anxiety (Section 2). A recent meta-analysis indicated that Glu levels are significantly elevated in the PFC of ASD patients [99], a brain region that often overlaps with the ACC in MRS studies. This Glu elevation in ASD patients could potentially mask the glutamatergic alterations observed in noncomorbid MDD. Thus, the observed discrepancy in Glu levels may be attributed to the homogeneity of the samples included in our study. After applying strict inclusion and exclusion criteria to eliminate psychiatric comorbidities, our study highlights the MDD-specific characteristics of neurotransmitter level changes.

The significantly decreased Glu levels in the ACC/PFC identified in our meta-analysis may reflect the hypothesis of E/I imbalance in the pathogenesis of MDD [14, 16]. The balance between GABAergic inhibition and glutamatergic excitation is essential for maintaining optimal PFC function [100]. In chronic stress-related affective disorders, such as MDD, the PFC often exhibits an over-inhibitory state, characterized by impaired glutamatergic activity and complex GABAergic dysregulation [101, 102], which may contribute to decreased Glu levels in the PFC of MDD patients. Given that this decrease in Glu levels can be reversed by the rapid-acting antidepressant ketamine/esketamine, the dysfunction of the Glu concentration may serve as a potential biomarker of disease status. Following subclinical dose of ketamine, increased Glu concentration have been observed in the PFC in both mice [103] and humans [104]. Further studies that use ketamine or esketamine as intervention strategies for MDD patients are needed. The OCC is involved in visual processing and plays an important role in language and reading. MDD patients present highly specific visual deficits with decreased GABA level in the visual perception region [71]. However, the relatively small sample size limits the generalizability of these findings. Our meta-analysis expanded the sample size and confirmed decreased GABA levels in the OCC. This finding is also consistent with a clinical trial [105] indicating that occipital bending, a physical asymmetry of the occipital lobes, may represent a meaningful biomarker in MDD.

We initially assumed that longitudinal 1H-MRS measurements would reveal treatment-induced alterations in neurometabolite concentrations, guided by prior evidence that traditional antidepressants could regulate Glu release and Glu receptors expression in vitro [12]. However, in our study, we did not find statistically robust differences between the pre and postintervention phases in MDD patients. This result might be explained by limited statistical power due to small sample sizes. On the other hand, this finding indicated possible heterogeneity in currently existing clinical studies. Heterogeneity may stem from two sources: Inter-study and inter-individual. The inter-study heterogeneity is derived from different treatment strategies: Some prescribed SSRIs with distinct dosages, drug types, and treatment courses [17, 70, 92–95, 98], whereas others apply physical therapies such as rTMS and ECT [77, 88, 91–93, 91–93, 96, 97]. The inter-individual heterogeneity might originate from the existence of MDD subgroups. First, the brain metabolic alterations differed among the physical therapy treatment-response subgroups. After physical therapy, patients with good treatment-responses exhibited significantly elevated GABA levels (6-weeks of rTMS treatment) [96] and Glx levels (ECT) [88] in the PFC, whereas the nonresponse group did not. Another possible subgroup characteristic concerning GABAergic/glutamatergic levels is sex. Though we did not find evidence for influence of sex on neurometabolic alterations in meta-regression, previous studies have found that the GABAergic systems of males and females responded differently to stress or depression in mice. After chronic stress exposure, female mice are more likely to exhibit depressive-like behaviors and increased parvalbumin-expressing GABAergic neurons [106, 107]. Additionally, previous studies have shown that MDD subtypes are divergent in their genetic components [108, 109], which may account for the variability in treatment responses, especially in TRD. Several genes encoding glutamatergic receptor subunits, which are implicated in the pathogenesis of TRD [110], may contribute to the nonsignificant changes in glutamatergic metabolites in some patients. These findings suggest that genetic heterogeneity within MDD subtypes could influence the metabolic response to treatment. Further studies could explore whether genetic variations related to glutamatergic/GABAergic function in specific MDD subgroups are associated with changes in cortical metabolic levels. In summary, while our analysis did not identify statistically significant differences between pre and postinterventional neurometabolic measures in MDD patients, our findings highlight the need for larger sample sizes to increase statistical power and facilitate homogeneous subgroup analyses. Such efforts could help determine whether glutamatergic metabolic changes in specific subgroups could serve as biomarkers of treatment response or predict drug efficacy, ultimately guiding personalized and early interventions.

## 5. Limitations

For comparison between the neurometabolites of MDD patients' pre and postintervention, this study was limited in its small sample size and heterogenouse intervention strategies. Therefore, our results, especially regarding no significant changes in neurometabolite levels between baseline and follow-up, still need more studies.

Notably, the *I*^2^ of our analysis is moderate-to-high, which suggestes that our results need to be considered with caution. However, the result was relatively stable after we performed a series of sensitivity analyses. Additionally, such heterogeneity is consiste with the assumption previously addressed by other studies [111] that biological subtypes may exist inside the large domain of MDD diagnosis. Second, MRS investigations of neurotransmitters face challenges such as low signal-to-noise ratio, especially when the filed strength is less than 3.0 T. This limitation can affect the accuracy of distinguishing Glu and Gln from Glx [112]. However, the majority of included studies were conducted at 3 T. Third, the present study included only patients with MDD, so it is not clear whether the identified changes are specific to patients with MDD or also occur in other psychiatric disorders. A recent meta-analysis reported a decreased Glu level in the mPFC (including the mPFC and ACC) in schizophrenia patients, which is consists with our findings in MDD patients [113]. Therefore, whether decreased Glu level in the brain represents a continuum bio-alteration in psychiatric disorders needs to be further investigated. Fourth, some of the patients with MDD included in the present study were not medication-naive patients; although we conducted subgroup analysis for antidepressant treatment therapy, the potential influence of pharmacological treatment on the findings cannot be ruled out.

## 6. Conclusion

In summary, the main findings of this meta-analysis are that MDD patients are characterized by regionally decreased glutamatergic neurotransmitter concentrations compared with healthy controls. The subgroup analysis showed that the decreased concentrations of glutamatergic neurotransmitters are independent of current treatment status (whether patients underwent drug or physical treatment at the time of MRS scanning or not). Additionally, we did not find significant changes in glutamatergic neurotransmitters in MDD patients related to antidepressant intervention. Changes in glutamatergic metabolites may be stable and intrinsic markers of MDD. Although this result was limited in sample size and high heterogeneity, it suggests the possibility of different biosubtypes of treatment response in MDD patients. Further research into the potential mechanisms of glutamatergic neurotransmitters in MDD could enhance our understanding of MDD pathogenesis and aid in identifying patient subgroups with different clinical prognoses. This, in turn, may facilitate the development of novel therapeutics.

## Figures and Tables

**Table 1:** The characteristics of studies for comparison between MDDs and controls.

Author (year)	MRI scanning parameter									Characteristic feature								Clinical symptoms assessment						Intervention	
										MDDs					Controls			HAMD-17	HAMD-24	MADRS	HAMA	Other		Pharmacotherapy	Physiotheraphy
	Channel	MRI type	Acquisiton sequnence	Field strength (*T*)	TE (ms)	TR (ms)	Spectral width (KHz)	CRLB threshold	Concentration scaling	*n*	Female (%)	Age (mean ± sd)	Overall course of disease	Untreated course of disease	*n*	Female (%)	Age (mean ± sd)	Baseline (mean ± sd)	Baseline (mean ± sd)	Baseline (mean ± sd)	Baseline (mean ± sd)	Assessment tool	Baseline (mean ± sd)		
Rebecca (2009)	8	1H	J editing	3	68	1500	5	—	Water	33	13 (39.39%)	38.3 ± 12.3	21.80 years	—	24	13 (54.17%)	37.25 ± 13.50	17.94 ± 5.50	—	—	19.44 ± 6.90	—	—	0	0

David (2004)	—	1H	PRESS	1.5	—	—	—	—	—	14	9 (64.29%)	15.63 ± 2.33	3.15 years	—	14	9 (40.91%)	15.47 ± 2.42	—	—	—	—	—	—	0	0

Bhagwagar (2008)	—	1H	PRESS and MEGA-PRESS	3	68	3000	2	＜20%	All metabolite concentrations were evaluated as ratios to creatine	12	8 (66.67%)	40.6 ± 4.2	—	Medication free	11	4 (36.36%)	34.3 ± 4.1	—	—	—	—	—	—	0	0

Binesh (2004)	—	1H	L-Cozy	1.5	30	2000	—	—	The metabolite ratios were calculated using the two-dimensional peak volumes with respect to the diagonal peak volume of total creatine at 3.0 ppm.	12	12 (100%)	72 ± 8	—	>2 weeks	16	16 (100%)	72 ± 7	—	—	—	—	—	—	0	0

Sanacora (2004)	—	1H	J editing	2.1	68	2000	—	—	GABA concentration was determined from the ratio of GABA–total creatine resonances: [GABA-] = (Area_GABA_/Area_Creatine_ − 0.07) x (0.93×1.01) x (3/2) x [Creatine], where creatine was 9 mmol/kg. Further details on the reference.	33	11 (33%)	41.87 ± 9.88	—	>2 weeks	38	23 (60.5%)	35.74 ± 11.40	—	—	—	—	MDRS	28.27	0	0

Menke (2012)	—	1H	PRESS	1.5	35	2000	—	—	Water	61	59.60%	51.1 ± 13.4	14.9 ± 12.1 years	—	50	50%	42.1 ± 14.3	—	—	—	—	BDI	15.2 ± 12.4	1	0

Nery (2009)	—	1H	PRESS	1.5	30	3000	2	<20%	Water	37	24 (64.90%)	36.6 ± 13.7	15.8 ± 12.3 years	Unmedicated	40	26 (65%)	40 ± 12.3	—	—	—	—	HAMD-21	14.8 ± 6.9	0	0

Wang (2016)	8	1H	MEGA-PRESS	3	68	2000	1	—	Water	19	19 (100.00%)	53.90 ± 2.56	0.50–2.00 years	>6.00 months	13	13 (100.00%)	52.62 ± 2.18	17.32 ± 1.73	—	—	15.42 ± 1.30	—	—	0	0

Horn (2010)	8	1H	PRESS	3	80	2000	1.2	<20%	All metabolite values are given as creatine ratios	18	8 (44.44%)	39.22 ± 12.67	—	—	22	10 (45.45%)	34.14 ± 6.67	—	—	—	—	HAMD-21	17.17 ± 4.74	1	0

Rosa (2017)	8	1H	PRESS	3	31	1500	2	<20%	Water	31	31 (100.00%)	27.7 ± 4.8	—	—	23	23 (100%)	29.0 ± 6.0	24.3 ± 5.7	—	—	—	—	—	1	0

McEwen (2012)	—	1 H	STEAM	3	240	3000	—	—	Water	12	12 (100.00%)	28.67 ± 7.45	—	≥3 months	12	12 (100%)	29.08 ± 4.89	—	—	—	—	—	—	0	0

Sanacora (1999)	—	1H	J editing	2.1	68	3390	1.5	—	The creatine signal was integrated over a 0.20-ppm bandwidth at 3.00 ppm in the GABA inverted spectrum. The following equation was used to calculate the GABA concentration: [GABA] = ([G*⁣*^*∗*^/Cr*⁣*^*∗*^]-[M/Cr*⁣*^*∗*^]) (ICF) (EE) (3/2) ([Cr]). Further details on the reference.	14	6 (42.86%)	42.9 ± 9.2	—	Medication free	18	7 (38.89%)	38.4 ± 10.2	—	—	—	—	HAMD-25	34 ± 7	0	0

Milne (2009)	—	1H	PRESS	3	35	2000	2.5	—	Water	14	10 (71.43%)	47.29 ± 10.59	24.43 ± 13.09 years	—	14	10 (71.43%)	41.79 ± 12.91	8.23 ± 5.64	—	—	—	BDI	12.62 ± 5.01	1	0

Draganov (2020)	8	1H	PRESS	3	38	2000	—	—	—	31	18 (58.06%)	37.29 ± 10.8	—	>2 weeks	63	53.12%	41.77 ± 10.1	22.81 ± 4.2	—	—	—	—	—	0	0

Wang (2019)	32	1H	MEGA-PRESS	3	68	1500	1.2	—	Water	19	19 (100.00%)	47.68 ± 1.86	—	Medication free	71	71 (100.00%)	46.80 ± 1.98	20.737 ± 2.579	—	—	—	—	—	0	0

Taylor (2015)	—	1H	Echo acquisition mode	7	10	3000	—	<10%	Water	16	10 (62.50%)	21.70 ± 3.30	28.60 months	—	16	5 (31.25%)	23.90 ± 4.70	12.40 ± 9.10	—	—	12.70 ± 10.90	—	—	1	0

Yuan (2011)	—	1H	PRESS	1.5	35	1500	—	—	The concentrations of NAA, Cho, mIns, and Glx were quantified relative to creatine by calculating the ratio of their respective AUCs to the AUC of creatine.	12	8 (66.67%)	41.60 ± 12.20	—	—	12	7 (58.33%)	42.50 ± 11.70	—	—	—	—	—	—	0	0

Rezende (2018)	8	1H	PRESS	3	31	1500	—	<20%	Metabolite levels were quantified and expressed as a ratio related to creatine concentration (/Cr).	20	20 (100.00%)	28.20 ± 4.80	—	—	19	19 (100%)	28.80 ± 4.30	—	—	—	—	EPDS	16.65 ± 6.18	0	0

Godlewska (2018)	32	1H	Semi-LASER pulse	7	32 (36 for OCC)	5000 (3000 for OCC)	—	<30%	Water	55	31 (56.36%)	31.3 ± 1.3	—	—	50	28 (56%)	31.3 ± 1.4	21.3 ± 0.7	—	—	—	BDI;STAI-S;SHAPS;CFS	BDI30.1 ± 1.1; STAI-S51.8 ± 1.6;SHAPS33.5 ± 0.9; CFS 22.4 ± 0.6	0	0

Zhang (2016)	32	1H	MEGA-PRESS	3	69	1500	—	<20%	Water	11	—	34.09 ± 8.78	—	—	11	—	33.64 ± 7.19	—	22.91 ± 3.02	—	11.09 ± 2.02	SDS;SAS	SDS 40.91 ± 5.99; SAS 38.00 ± 9.03	1	0

Kahl (2020)	12	1H	EPSI	3	17.6	1550	—	<25%	Water	32	22 (68.70%)	36.8 ± 12.6	—	—	32	22 (68.7%)	37 ± 12.4	—	—	—	—	BDI-2	20.9 ± 10.9	1	0

Li (2014)	8	1H	PRESS	3	80	With water suppression: 2000; without water suppression: 10,000	1.2	<20%	Creatine + phosphocreatine	24	12 (50.00%)	36.87	—	—	25	12 (48%)	37.05	18 ± 5	—	—	—	—	—	1	0

Ma (2008)	—	1H	PRESS	1.5	135	2000	—	—	Creatine	23	11 (47.83%)	35.61 ± 14.02	11.85	—	23	11 (47.83%)	34.78 ± 13.64	—	—	—	—	—	—	0	0

Zhao (2015)	—	1H	Multivoxel 3D-CSI	1.5	135	1700	—	—	Creatine	30	21 (70.00%)	—	—	—	30	21 (70.00%)	—	—	23.60 ± 3.90	—	—	—	—	0	0

Poletti (2016)	—	1H	PRESS	3	30	2000	—	—	Creatine + phosphocreatine	19	14 (73.68%)	51.15 ± 11.31	18.74 ± 9.61 years	—	17	11 (64.71%)	26.53 ± 8.26	—	—	—	—	IDSC; RFQ	IDSC30.47 ± 13.41;RFQ28.58 ± 9.24	1	0

Deligiannidis (2019)	—	1H	MEGA-PRESS	3	68	2000	—	—	Creatine	23	23 (100.00%)	28.6 ± 4.9	—	—	28	28(100%)	29.0 ± 5.0	14.1 ± 6.1	—	—	18.1 ± 7.7	Edinburgh Postnatal Depression Scale (EPDS); Spielberger State-Trait Anxiety (STAI-S); Sheehan Disability Scale (SDS)	EPDS12.8 ± 4.0; STAI-S44.2 ± 10.0;SDS11.7 ± 6.1	0	0

Hasler (2007)	—	1H	PRESS-based J editing	3	68	1500	5	—	The concentrations of GABA, choline, NAA, and coedited Glx (ie., glutamate and glutamine) are expressed in millimoles per liter referenced to the concentration of creatine that was set at 93 mg/dL (7100 µmol/L)	20	13 (65.00%)	34 ± 11.2	18.8 ± 13.5 years	41 ± 61 months	20	13 (65%)	34.8 ± 12.4	—	—	27 ± 4.3	—	HAMD	22 ± 6	0	0

Michael (2003)	—	1H	STEAM	1.5	20	2500	—	—	—	12	8 (66.67%)	63.40 ± 10.60	—	3–8 days	12	6 (50.00%)	62.00 ± 8.70	—	—	37.90 ± 9.60	—	—	—	0	0

Shirayama (2016)	12	1H	PRESS	3	30	4000	—	6.3 for glu/5.7 for Glx/14.4 for gln	—	22	5 (22.73%)	40.90 ± 2.40	—	—	27	9 (33.33%)	36.80 ± 8.40	21.50 ± 3.90	—	—	—	—	—	0	0

Godlewska (2015)	32	1H	SPECIAL pulse	3	8500	3200	—	<20%	Total creatine (creatine + phosphocreatine)	33	19 (57.58%)	29.9 ± 10.6	—	—	27	16 (59.26%)	30.3 ± 10.6	22.3 ± 4.6	—	—	—	BDI	30.1 ± 6.4	0	0

Smith (2021)	32	H	STEAM	7	14/15	3000	3	—	Levels of GABA, Glu, GSH, NAAG, NAA, and myo-inositol were quantified relative to total creatine (creatine + phosphocreatine)	9	5 (55.56%)	70.00 ± 7.00	—	1 years	9	4 (44.4%)	67.00 ± 7.00	—	17.00 ± 2.00	—	—	BDI	22.00 ± 8.00	0	0

Taylor (2009)		1H	PRESS	3	26	3000	—	—	Creatine	14	10 (71.43%)	32.6	—	—	16	11 (68.75%)	31.8	—	—	—	—	BDI	2.70 ± 3.10	0	0
													—	—								HDRS	0.30 ± 0.50		

Song (2021)	32	1H	STEAM	7	6	7100	4	<20%	Water	18	10 (55.56%)	22.80 ± 4.10	—	—	20	10 (50.00%)	23.40 ± 2.10	23.80 ± 3.60	—	—	—	—	—	1	0

Rosenberg (2005)	—	1H	PRESS	1.5	30	3000	2.5	—	—	14	9 (64.29%)	15.63 ± 2.33	37.82	—	14	9 (64.29%)	15.47 ± 2.42	—	—	—	—	CDRS-R	55.50 ± 8.59	0	0

Kantrowitz (2021)	—	1H	PROBE-J/PROBE-P	3	68/80	1500/2000	—	—	We report results using creatine as a normalization reference	34	22 (64.71%)	37.2 ± 10.7	9.4 ± 13.3 years	≥2 weeks	32	41%	35.1 ± 9.6	—	—	30.7 ± 3.5	—	—	—	0	0

Shaw (2013)	—	1H	MEGA-PRESS	3	68	1800	—	—	Water	19	19 (100%)	23.00 ± 2.60	—	—	18	18 (100%)	21.00 ± 1.50	—	—	—	—	BDI	5.70 ± 3.40	0	0

Li (2016)	8	H	PRESS	3	30	1500	—	≤20%	External standard phantom was used to calibrate the metabolite absolute concentrations	20	13 (65%)	28 ± 9.1	56.4 ± 29.4	≥2 weeks	20	10 (50%)	31.7 ± 11.4	26.5 ± 7.1	—	—	—	—	—	0	0

Persson (2021)	32	1H	MEGA-PRESS	3	68	2000	2	—	Quantify dACC GABA + and Glu (contrasted against total creatine)	42	21 (50%)	29 ± 9.4	—	—	45	27 (60%)	29.5 ± 11.2	—	—	29.6 ± 7.7	—	—	—	1	0

Venkatraman (2009)	—	1H	PRESS	3	30	3000	—	—	Water	14	8 (57.20%)	72.10 ± 4.60	Mean age of onset 49.5 years	—	12	6 (50.00%)	72.70 ± 5.30	—	—	6.3 ± 5.6	—	—	—	1	0

Block (2009)	—	1H	PRESS	3	140/30	2000	2	—	Creatine	18	8 (44.44%)	36 ± 10	—	≥8 weeks	10	4 (40%)	36 ± 19	—	—	—	—	—	—	0	0

Bonnekoh (2023)	64	1H	PRESS	3	35	6000	1.25	—	Total creatine (creatine and phosphocreatine) concentration	22	11 (50.00%)	31.90 ± 10.80	5.9 years	—	23	10 (43.48%)	33.80 ± 11.70	—	—	—	—	HAMD-21; BDI-Ⅱ	HAMD-21 18.80 ± 7.70	1	0

Kong (2023)	8	1H	PRESS	3	29	3500	—	—	—	30	17 (56.67%)	32.20 ± 11.80	48.60 months	—	20	10 (50%)	29.00 ± 8.30	—	—	—	—	HDRS	20.70 ± 4.90	0	0

Ritter (2022)	8	1H	MEGA-PRESS	3	69	2000	—	—	Water	98	75 (76.53%)	24.43 ± 4.78	—	—	251	153 (60.96%)	24.92 ± 4.47	—	—	—	—	HAMD-21	6.20 ± 5.00	0	0

Wang (2022)	8	1H	PRESS	3	30	2000	5	—	—	15	13 (86.67)	26.07 ± 9.77	—	—	10	8 (80.00%)	27.20 ± 6.51	—	—	—	—	ACIPS	72.27 ± 14.43	1	0

Baeken (2017)	—	1H	PRESS	3	40	2000	—	—	Water	18	12 (66.67%)	47.17 ± 12.54	3.07 years	2 weeks washout	18	12 (66.67%)	45.83 ± 12.34	—	—	—	—	BDI-Ⅱ	3.50 ± 3.62	0	0

Portella (2011)	8	1H	SVS-PRESS	3	38	2000	—	—	Water	19	15 (78.95%)	50.95 ± 7.30	27.84 years	—	15	10 (66.67%)	40.47 ± 11.60	—	—	—	—	HDRS	21.39 ± 4.50	1	0

Yang (2016)	24	1H	PRESS	3	30	2000	—	—	Water	17	9 (52.94%)	18.00 ± 1.92	—	—	11	8 (72.73%)	19.31 ± 1.30	—	—	—	22.71 ± 9.52	HAMD-21	23.94 ± 7.78	1	0
																BDI						—			
																—						73.94 ± 10.35			

*Note:* Author (year), the name of the first author with publication year. CDRS, Children's Depression Rating Scale-Revised; TE, echo time; TR, repetition time.

Abbreviations: ACIPS, Anticipatory and Consummatory Interpersonal Pleasure Scale; BDI, Beck Depression Inventory; CFS, Clinical Frailty Scale; CRLB, Cramér Rao Lower Bounds; EPDS, Edinburgh Postnatal Depression Scale; HAMD, Hamilton Depression Rating Scale; MADRS, Montgomery–Åsberg Depression Rating Scale; MDD, major depressive disorder; MRI, magnetic resonance imaging; PRESS, point resolved spectroscopy; RFQ, reflective functioning questionary; SAS, Self-Rating Anxiety Scale; SDS, Self-Rating Depression Scale; SHAPS, Snaith–Hamilton Pleasure Scale; STAIS, the state-trait anxiety inventory; STEAM, stimulated echo acquisition mode.

**Table 2:** Pooled effect sizes of region-specific neurometabolites for comparison between patients and controls.

Random effect model	Study number	Patients (*n*)	Controls (*n*)	SMD (95% CIs)	*p*-Value	*I* ^2^ (%)	*Q*
Glx							—
ACC	21	410	428	−0.33 (−0.56; −0.09)	0.006*⁣*^*∗*^	61	51.40
PFC	17	363	385	−0.35 (−0.60; −0.10)	0.006*⁣*^*∗*^	62	42.27
Hippocampus	6	165	144	−0.39 (−0.80; 0.02)	0.06	61	12.66
Glu							—
ACC	18	400	415	−1.10 (−2.14; −0.06)	0.04*⁣*^*∗*^	90	175.05
PFC	12	358	529	−0.33 (−1.22; 0.56)	0.46	97	429.49
OCC	5	149	145	−0.31 (−1.22; 0.61)	0.51	94	66.30
Gln							—
ACC	5	122	117	0.61 (−0.50; 1.73)	0.28	95	76.04
OCC	4	116	107	−0.42 (−0.94; 0.09)	0.11	73	11.10
GABA							—
ACC	11	223	302	−0.24 (−0.60; 0.11)	0.18	76	41.81
PFC	9	262	442	−0.77 (−2.05; 0.52)	0.24	98	460.42
OCC	7	173	173	−0.70 (−1.21; −0.19)	0.007*⁣*^*∗*^	75	23.88

*Note:* Glx, glutamine + glutamate; *p*-Value, for describing the significance of the pooled effect according to random-effects model; *I*^2^, An index of heterogeneity; *Q*, the Cochran's *Q* statistic for heterogeneity.

Abbreviations: ACC, anterior cingulate cortex; CI, confidence intervals; GABA, γ-aminobutyric acid; Glu, glutamate; OCC, occipital cortex; PFC, prefrontal cortex; SMD, standardized mean difference.

*⁣* ^*∗*^Asterisk marks significant results with *p*-value less than 0.05.

**Table 3:** Pooled effect sizes of region-specific neurometabolites for comparison between baseline and follow-up measurement in patients.

Random effect model	Study number	Baseline (*n*)	Follow-up (*n*)	SMD (95% CI)	*p*-Value	*I* ^2^ (%)	*Q*
Glx							—
ACC	8	161	161	0.004 (−0.28; 0.29)	0.98	33.8	10.57
PFC	7	92	92	−0.15 (−0.47; 0.17)	0.36	21.5	7.65
Glu							—
ACC	4	41	41	0.09(−0.34; 0.53)	0.68	0.0	1.28
PFC	6	67	67	0.13(−0.27; 0.53)	0.53	19.4	6.20
GABA							—
ACC	5	126	126	0.02 (−0.30; 0.35)	0.22	31.0	5.78
PFC	5	68	68	−0.23 (−0.73; 0.28)	0.38	50.2	8.03

*Note:* Glx, glutamine + glutamate; GABA, *γ*-aminobutyric acid; *p*-Value, for describing the significance of the pooled effect according to random-effects model; *I*^2^, An index of heterogeneity; *Q*, the Cochran's Q statistic.

Abbreviations: ACC, anterior cingulate cortex; CI, confidence intervals; Glu, glutamate; OCC, occipital cortex; PFC, prefrontal cortex; SMD, standardized mean difference.

## Data Availability

Data sharing is not applicable to this article as no new data were created or analyzed in this study.

## Supporting Information

Supporting Information Supporting information Table S1: Descriptions for articles including comorbid anxiety disorders. Supporting information Table S2: The characteristics of studies for comparison between baseline and follow-up measurement in patients. Supporting information Table S3: Meta-analysis pooled effect size before and after outliers' removal for comparison between MDD patients and controls. Supporting information Table S4: Results of subgroup analysis on MRI scanning parameter of comparison between patients and controls. Supporting information Table S5: Results of subgroup analysis on under treatment or not of comparison between MDD patients and controls. Supporting information Table S6: Results of meta-regression for MDD patients and controls. Supporting information Table S7: The Egger test for publication bias. Supporting information Table S8: Meta-analysis pooled effect size before and after outliers' removal for comparison MDD patients at baseline and intervention. Supporting information Table S9: Results of meta-regression for MDD patients at baseline and intervention. Supporting information Figure S1: Effect sizes of neurometabolites in brain regions that did not represent significant differences between MDD and healthy controls. Supporting information Figure S2: Effect sizes of neurometabolites in brain regions between patients pre and postintervention. Supporting information Figure S3: Funnel plots for comparison between patients and controls with study size above ten and I2 less than 75%. Supporting information Figure S4: Doi plot analysis and LFK index of publication bias for comparison in patients pre and postintervention below ten and I2 less than 75%. Supporting information Figure S5: Doi plot analysis and LFK index of publication bias for comparison between patients and controls with study size below ten and I2 less than 75%.
