# Supplementary material for: A Meta-Analysis of Magnetic Resonance Spectroscopy Studies on Glutamatergic Neurometabolite Levels in Major Depressive Disorder
Source: Depress Anxiety. 2025 Jul 31;2025:5180077. doi: 10.1155/da/5180077 (PMC12331408; doi:10.1155/da/5180077)
Supplement: Supporting Information — Table S1: Descriptions for articles including comorbid anxiety disorders. Supporting information Table S2: The characteristics of studies for comparison between baseline and follow-up measurement in patients. Supporting information Table S3: Meta-analysis pooled effect size before and after outliers' removal for comparison between MDD patients and controls. Supporting information Table S4: Results of subgroup analysis on MRI scanning parameter of comparison between patients and controls. Supporting information Table S5: Results of subgroup analysis on under treatment or not of comparison between MDD patients and controls. Supporting information Table S6: Results of meta-regression for MDD patients and controls. Supporting information Table S7: The Egger test for publication bias. Supporting information Table S8: Meta-analysis pooled effect size before and after outliers' removal for comparison MDD patients at baseline and intervention. Supporting information Table S9: Results of meta-regression for MDD patients at baseline and intervention. Supporting information Figure S1: Effect sizes of neurometabolites in brain regions that did not represent significant differences between MDD and healthy controls. Supporting information Figure S2: Effect sizes of neurometabolites in brain regions between patients pre and postintervention. Supporting information Figure S3: Funnel plots for comparison between patients and controls with study size above ten and I2 less than 75%. Supporting information Figure S4: Doi plot analysis and LFK index of publication bias for comparison in patients pre and postintervention below ten and I2 less than 75%. Supporting information Figure S5: Doi plot analysis and LFK index of publication bias for comparison between patients and controls with study size below ten and I2 less than 75%. [file 5180077.f1.docx]

SUPPLEMENTARY MATERIAL

**Table of contents**

**Supplementary Tables**

Supplementary Table1: Descriptions for articles including comorbid anxiety disorders (See Excel).

Supplementary Table 2: The characteristics of studies for comparison between baseline and follow-up measurement in patients (See Excel).

Supplementary Table 3: Meta-analysis pooled effect size before and after outliers’ removal for comparison between MDD patients and controls.

Supplementary Table 4: Results of subgroup analysis on MRI scanning parameter of comparison between patients and controls.

Supplementary Table 5: Results of subgroup analysis on under treatment or not of comparison between MDD patients and controls.

Supplementary Table 6: Results of meta-regression for MDD patients and controls.

Supplementary Table 7: The Egger test for publication bias.

Supplementary Table 8: Meta-analysis pooled effect size before and after outliers’ removal for comparison MDD patients at baseline and intervention.

Supplementary Table 9: Results of meta-regression for MDD patients at baseline and intervention.

**Supplementary Figures**

Supplementary Figure 1: Effect sizes of neurometabolites in brain regions that did not represent significant differences between MDD and healthy controls.

Supplementary Figure 2: Effect sizes of neurometabolites in brain regions between patients pre- and post-intervention.

Supplementary Figure 3: Funnel plots for comparison between patients and controls with study size above ten and I^2^ less than 75%.

Supplementary Figure 4: Doi plot analysis and LFK index of publication bias for comparison in patients pre- and post-intervention below ten and I^2^ less than 75%.

Supplementary Figure 5: Doi plot analysis and LFK index of publication bias for comparison between patients and controls with study size below ten and I^2^ less than 75%.

**Supplementary Table 3. Meta-analysis pooled effect size before and after outliers’ removal for comparison between MDD patients and controls**

| Random effect model | Results for random effects model | | Test of heterogeneity | | Removed studies |
| --- | --- | --- | --- | --- | --- |
|  |  | |  | |  |
|  | Analysis | SMD (95%CI) | I^2^ | *p-*value for Q test |  |
| **Glx** |  |  |  |  |  |
| ACC | Main analysis | -0.33 (-0.56; -0.09) * | 61.1% | 0.0001 | - |
|  | Infl. Cases Removed | -0.27(-0.42; -0.12) * | 0.0% | 0.4756 | Bhagwagar 2008  David 2004  Kantrowitz 2021 |
| PFC | Main analysis | -0.35 (-0.60; -0.10) * | 62.2% | 0.0004 | - |
|  | Infl. Cases Removed | -0.42 (-0.62; -0.21) * | 43.9% | 0.0311 | Kantrowitz 2021 |
| hippocampus | Main analysis | -0.39 (-0.80; 0.02) | 60.5% | 0.0268 |  |
|  | Infl. Cases Removed | - | - | - | No outliers detected |
| **Glu** |  |  |  |  |  |
| ACC | Main analysis | -1.10 (-2.14; -0.06) * | 90.3% | < 0.0001 | - |
|  | Infl. Cases Removed | -0.47 (-0.83; -0.12) * | 74.8% | < 0.0001 | McEwen 2012  Horn 2010  Kong 2023 |
| PFC | Main analysis | -0.33 (-1.22; 0.56) | 97.4% | < 0.0001 | - |
|  | Infl. Cases Removed | -0.01 (-0.31; 0.28) | 53.6% | 0.0278 | Kahl 2020  Ritter 2022  Portella 2011 |
| OCC | Main analysis | -0.31 (-1.22; 0.61) | 94.0% | <0.0001 |  |
|  | Infl. Cases Removed | - | - | - | No outliers detected |
| **Gln** |  |  |  |  |  |
| ACC | Main analysis | 0.61 (-0.50; 1.73) | 94.7% | <0.0001 |  |
|  | Infl. Cases Removed | 0.02 (-0.45; 0.49) | 44.2% | 0.1464 | Godlewska 2018 |
| OCC | Main analysis | -0.42 (-0.94; 0.09) | 73.0% | 0.0112 |  |
|  | Infl. Cases Removed | - | - | - | No outliers detected |
| **GABA** |  |  |  |  |  |
| ACC | Main analysis | -0.24 (-0.60; 0.11) | 76.1% | < 0.0001 | - |
|  | Infl. Cases Removed | -0.35 (-0.68; -0.01) * | 66.0% | 0.0017 | Draganov 2020 |
| PFC | Main analysis | -0.77 (-2.05; 0.52) | 98.3% | <0.0001 | - |
|  | Infl. Cases Removed | -0.13 (-0.56; 0.31) | 76.1% | 0.0001 | Ritter 2022 |
| OCC | Main analysis | -0.70 (-1.21; -0.19) * | 74.9% | 0.0005 |  |
|  | Infl. Cases Removed | -0.50 (-0.84; -0.15) * | 54.9% | 0.0498 | Sanacora 1999 |

Notes: *SMD*, Standardized mean difference; *CI*, Confidence intervals; *ACC*, anterior cingulate cortex; *PFC*, prefrontal cortex; *OCC*, occipital cortex; *Glu*, glutamate; *Glx*, glutamine + glutamate; *GABA*, γ-aminobutyric acid; *I^2^*, an index of heterogeneity; *Q*, the Cochran's Q statistic tests for heterogeneity; ^*^: *p*-value < 0.05.

**Supplementary Table 4. Results of subgroup analysis on MRI scanning parameter of comparison between patients and controls**

| Random effect model | Results for subgroups (random effects model) | | | | Test for subgroup differences | |
| --- | --- | --- | --- | --- | --- | --- |
|  |  | | | |  | |
|  | subgroup | study size | SMD (95%CI) | I^2^ | Q | *p*-value |
| **Glx** |  |  |  |  |  |  |
| ACC | Cr | 8 | -0.38 (-0.88; 0.12) | 75.9% | 0.49 | 0.78 |
|  | Water | 8 | -0.25 (-0.52; 0.02) | 28.4% |  |  |
|  | Na | 5 | -0.44 (-0.96; 0.09) | 65.9% |  |  |
| PFC | Cr | 6 | -0.18 (-0.66; 0.30) | 73.0% | 1.41 | 0.49 |
|  | Water | 9 | -0.41 (-0.75; -0.07) | 58.1% |  |  |
|  | Na | 2 | -0.54 (-0.90; -0.18) | 0.0% |  |  |
| hippocampus | Cr | 4 | -0.56(-1.08; -0.04) | 60.3% | 1.00 | 0.32 |
|  | Water | 2 | -0.07(-0.87; 0.75) | 74.4% |  |  |
| **Glu** |  |  |  |  |  |  |
| ACC | Cr | 7 | -1.79 (-4.56; 0.99) | 90.8% | 1.89 | 0.39 |
|  | Water | 8 | -0.39 (-1.12; 0.35) | 85.2% |  |  |
|  | Na | 3 | -2.05 (-5.08; 0.98) | 96.3% |  |  |
| PFC | Cr | 1 | 0.19 (-0.29; 0.68) | - | 4.07 | 0.13 |
|  | Water | 10 | -0.38 (-1.45; 0.70) | 97.8% |  |  |
|  | Na | 1 | -0.42 (-0.85; 0.02) | - |  |  |
| OCC | Cr | 3 | -1.37 (-1.91; -0.82) | 47.0% | 16.69 | <0.001* |
|  | Water | 2 | 0.41 (-0.25; 1.07) | 73.5% |  |  |
| **Gln** |  |  |  |  |  |  |
| ACC | Cr | 1 | -0.47 (-1.04; 0.10) | - | 4.19 | 0.12 |
|  | Water | 2 | 1.43 (-1.08; 3.93) | 96.9% |  |  |
|  | Na | 2 | 0.29 (-0.42; 1.01) | 40.3% |  |  |
| OCC | Cr | 2 | 0.02 (-0.42; 0.47) | 0.0% | 5.91 | 0.01* |
|  | Water | 2 | -0.77 (-1.24; -0.30) | 37.9% |  |  |
| **GABA** |  |  |  |  |  |  |
| ACC | Cr | 5 | -0.17 (-0.56; 0.23) | 59.6% | 5.76 | 0.06 |
|  | Water | 4 | -0.66 (-1.25; -0.07) | 65.4% |  |  |
|  | Na | 2 | 0.39 (-0.23; 1.01) | 49.8% |  |  |

Notes: *SMD*, Standardized mean difference; *CI*, Confidence intervals; *ACC*, anterior cingulate cortex; *PFC*, prefrontal cortex; *OCC*, occipital cortex; *Glu*, glutamate; *Glx*, glutamine + glutamate; *GABA*, γ-aminobutyric acid; *Cr*, subgroup with the creatine or total creatine levels as the MRS reference; *Water*, subgroup with water levels as the MRS reference; *Na*, subgroup without the MRS reference information; *I^2^*, An index of heterogeneity; *Q*, the Q statistic tests for subgroup differences based on random effects model, and Q-test with *p*-value less than 0.05 was considered significant; ^*^: *p*-value < 0.05.

**Supplementary Table 4. Results of subgroup analysis on MRI scanning parameter of comparison between patients and controls (continued)**

| Random effect model | Results for subgroups (random effects model) | | | | Test for subgroup differences | |
| --- | --- | --- | --- | --- | --- | --- |
|  |  | | | |  | |
|  | subgroup | Study size | SMD (95%CI) | I^2^ | Q | *p*-value |
| PFC | Cr | 3 | -0.21 (-0.91; 0.49) | 74.3% | 6.50 | 0.04* |
|  | water | 5 | -1.40 (-3.59; 0.79) | 98.8% |  |  |
|  | Na | 1 | 0.63 (0.19; 1.07) | - |  |  |
| OCC | Cr | 4 | -0.77 (-1.59; 0.05) | 80.1% | 0.05 | 0.83 |
|  | water | 3 | -0.64 (-1.43; 0.14) | 77.0% |  |  |

Notes: *SMD*, Standardized mean difference; *CI*, Confidence intervals; *ACC*, anterior cingulate cortex; *PFC*, prefrontal cortex; *OCC*, occipital cortex; *Glu*, glutamate; *Glx*, glutamine + glutamate; *GABA*, γ-aminobutyric acid; *Cr*, subgroup with the creatine or total creatine levels as the MRS reference; *Water*, subgroup with water levels as the MRS reference; *Na*, subgroup without the MRS reference information; *I^2^*, An index of heterogeneity; *Q*, the Q statistic tests for subgroup differences based on random effects model, and Q-test with *p*-value less than 0.05 was considered significant; ^*^: *p*-value < 0.05.

**Supplementary Table 5. Results of subgroup analysis on under treatment or not of comparison between MDD patients and controls**

| Random effect model | Results for subgroups (random effects model) | | | | Test for subgroup differences | |
| --- | --- | --- | --- | --- | --- | --- |
|  |  | | | |  | |
|  | subgroup | Study size | SMD (95%CI) | I^2^ | Q | *p*-value |
| **Glx** |  |  |  |  |  |  |
| ACC | 0 | 11 | -0.46 (-0.87; -0.06) | 74.9% | 1.32 | 0.25 |
|  | 1 | 10 | -0.19 (-0.41; 0.03) | 15.6% |  |  |
| PFC | 0 | 10 | -0.25 (-0.57; 0.06) | 66.9% | 1.02 | 0.31 |
|  | 1 | 7 | -0.51 (-0.90; -0.12) | 49.7% |  |  |
|  |  |  |  |  |  |  |
| hippocampus | 0 | 2 | -0.55(-1.00; -0.10) | 23.7% | 0.37 | 0.54 |
|  | 1 | 4 | -0.30(-0.97; 0.36) | 71.4% |  |  |
| **Glu** |  |  |  |  |  |  |
| ACC | 0 | 9 | -1.03 (-2.01; -0.05) | 89.5% | 0.05 | 0.82 |
|  | 1 | 9 | -1.31 (-3.51; 0.88) | 89.5% |  |  |
| PFC | 0 | 5 | -0.88 (-2.34; 0.58) | 98.6% | 1.06 | 0.30 |
|  | 1 | 7 | 0.08 (-1.02; 1.18) | 92.6% |  |  |
| OCC | 0 | 3 | -0.53(-1.63; 0.58) | 92.7% | 0.21 | 0.64 |
|  | 1 | 2 | 0.006(-1.98; 1.99) | 95.5% |  |  |
| **Gln** |  |  |  |  |  |  |
| ACC | 0 | 4 | 0.59 (-0.83; 2.01) | 96.1% | 0.01 | 0.91 |
|  | 1 | 1 | 0.68 (-0.14; 1.51) | - |  |  |
| OCC | 0 | 3 | -0.40 (-1.10; 0.29) | 82.0% | 0.01 | 0.91 |
|  | 1 | 1 | -0.46 (-1.10; 0.19) | - |  |  |
| **GABA** |  |  |  |  |  |  |
| ACC | 0 | 8 | -0.37 (-0.82; 0.09) | 81.2% | 3.02 | 0.08 |
|  | 1 | 3 | 0.14 (-0.20; 0.48) | 0.0% |  |  |
| PFC | 0 | 8 | -0.87 (-2.30; 0.56) | 98.5% | 1.25 | 0.26 |
|  | 1 | 1 | 0.08 (-0.76; 0.91) | - |  |  |
| OCC | 0 | 4 | -0.30 (-0.58; -0.02) | 8.9% | 5.98 | 0.01 |
|  | 1 | 3 | -1.35 (-2.14; -0.56) | 72.4% |  |  |

Notes: *0*, subgroup without any drug or physical treatment or with a washout period when undertaken the examine; *1*, subgroup received treatment when undertaken the examine; *SMD*, Standardized mean difference; *CI*, Confidence intervals; *ACC*, anterior cingulate cortex; *PFC*, prefrontal cortex; *OCC*, occipital cortex; *Glu*, glutamate; *Glx*, glutamine + glutamate; *GABA*, γ-aminobutyric acid; *I^2^*, An index of heterogeneity; *Q*, the Q statistic tests for subgroup differences based on random effects model, and Q-test with *p*-value less than 0.05 was considered significant; ^*^: *p*-value < 0.05.

**Supplementary Table 6. Results of meta-regression for MDD patients and controls**

| Random effect model | Test for coefficient | | | | Test for model | | | |
| --- | --- | --- | --- | --- | --- | --- | --- | --- |
|  | Predictor | Coefficient (95%CI) | Z-value | *p*-value | R^2^ | | Q_M_-value | *p*-value |
| **Glx** |  |  |  |  |  | |  |  |
| ACC | Patients’ age | -0.002 (-0.02, 0.02) | -0.22 | 0.83 | 0.00% | | 0.05 | 0.8278 |
|  | Patients’ gender  (female ratio) | -0.0025 (-0.01, 0.01) | -0.44 | 0.66 | 0.00% | | 0.19 | 0.6620 |
|  | Patients’  sample size | 0.03 (0.002, 0.06) | 2.35 | 0.02 * | 26.14% | | 4.41 | 0.04 * |
| PFC | Patients’ age | -0.003 (-0.02, 0.02) | -0.34 | 0.73 | 0.00% | | 0.12 | 0.73 |
|  | Patients’ gender  (female ratio) | 0.004 (-0.01, 0.02) | 0.58 | 0.56 | 0.00% | | 0.34 | 0.56 |
|  | Patients’  sample size | 0.02 (-0.003, 0.05) | 1.72 | 0.09 | 23.88% | | 2.95 | 0.09 |
| hippocampus | Patients’ age | 0.06 (-0.004, 0.12) | 1.82 | 0.07 | 45.73% | | 3.30 | 0.07 |
|  | Patients’ gender  (female ratio) | 0.04 (0.01, 0.06) | 3.15 | 0.002* | 100.00% | | 9.90 | 0.002* |
|  | Patients’  sample size | -0.003 (-0.03, 0.03) | -0.22 | 0.82 | 0.00% | | 0.05 | 0.82 |
| **Glu** |  |  |  |  |  | |  |  |
| ACC | Patients’ age | -0.007(-0.08, 0.07) | -0.18 | 0.86 | 0.00% | | 0.03 | 0.86 |
|  | Patients’ gender  (female ratio) | 0.029 (-0.027, 0.08) | 1.01 | 0.31 | 0.00% | | 1.02 | 0.31 |
|  | Patients’  sample size | 0.02 (-0.07, 0.11) | 0.40 | 0.68 | 0.00% | | 0.16 | 0.68 |
| PFC | Patients’ age | -0.0005 (-0.07 0.07) | -0.01 | 0.99 | 0.00% | | 0.0002 | 0.99 |
|  | Patients’ gender  (female ratio) | -0.0025(-0.05 0.05) | -0.10 | 0.92 | 0.00% | | 0.01 | 0.92 |
|  | Patients’  sample size | -0.04 (-0.07, -0.008) | -2.42 | 0.01* | 33.41% | | 5.87 | 0.01 |
| OCC | Patients’ age | 0.10 (-0.04, 0.25) | 1.40 | 0.16 | 25.50% | | 1.96 | 0.16 |
|  | Patients’ gender  (female ratio) | -0.07 (-0.16, 0.02) | -1.59 | 0.11 | 35.61% | | 2.54 | 0.11 |
|  | Patients’  sample size | -0.02 (-0.08, 0.04) | -0.64 | 0.52 | 0.00% | | 0.41 | 0.52 |
| **Gln** |  |  |  |  |  |  |  |  |
| ACC | Patients’ age | 0.007 (-0.14, 0.16) | 0.09 | 0.92 | 0.00% | | 0.009 | 0.92 |
|  | Patients’ gender  (female ratio) | 0.01 (-0.05, 0.07) | 0.47 | 0.64 | 0.00% | | 0.22 | 0.64 |
|  | Patients’  sample size | 0.06 (0.03, 0.10) | 3.40 | <0.001* | 0.00% | | 11.54 | < 0.001* |
| OCC | Patients’ age | -0.01 (-0.25, 0.23) | -0.11 | 0.91 | 0.00% | | 0.01 | 0.91 |

**Supplementary Table 6. Results of meta-regression for MDD patients and controls (continued)**

| Random effect model | Test for coefficient | | | | Test for model | | |  |
| --- | --- | --- | --- | --- | --- | --- | --- | --- |
|  | Predictor | Coefficient (95%CI) | Z-value | *p*-value | R^2^ | Q_M_-value | *p*-value |  |
|  | Patients’ gender  (female ratio) | 0.44 (-0.20, 1.08) | 1.35 | 0.17 | 39.90% | 1.84 | 0.17 | |
|  | Patients’  sample size | -0.01 (-0.04, 0.02) | -0.81 | 0.42 | 0.00% | 0.65 | 0.42 | |
| **GABA** |  |  |  |  |  |  |  | |
| ACC | Patients’ age | -0.02 (-0.05, 0.005) | -1.64 | 0.10 | 22.87% | 2.70 | 0.10 | |
|  | Patients’ gender  (female ratio) | -0.02 (-0.03, 0.01) | -1.84 | 0.06 | 27.81% | 3.40 | 0.06 | |
|  | Patients’  sample size | 0.01 (-0.02, 0.05) | 0.66 | 0.51 | 0.00% | 0.44 | 0.51 | |
| PFC | Patients’ age | 0.04 (-0.04, 0.13) | 0.98 | 0.33 | 0.00% | 0.96 | 0.33 | |
|  | Patients’ gender  (female ratio) | -0.00 (-0.09, 0.09) | -0.02 | 0.98 | 0.00% | 0.0007 | 0.98 | |
|  | Patients’  sample size | -0.07 (-0.09, -0.05) | -6.69 | <0.001* | 86.20% | 44.81 | < 0.001* | |
| OCC | Patients’ age | -0.04 (-0.10, 0.02) | -1.24 | 0.21 | 5.67% | 1.53 | 0.21 | |
|  | Patients’ gender  (female ratio) | 0.003 (-0.02, 0.02) | 0.27 | 0.79 | 0.00% | 0.07 | 0.79 | |
|  | Patients’  sample size | 0.04 (-0.03, 0.11) | 1.12 | 0.26 | 0.00% | 1.26 | 0.26 | |

Notes: *CI*, Confidence intervals; *ACC*, anterior cingulate cortex; *PFC*, prefrontal cortex; *OCC*, occipital cortex; *Glu*, glutamate; *Glx*, glutamine + glutamate; *GABA*, γ-aminobutyric acid; *Coefficient (95% CI)*, the estimated change in the effect size per one-unit increase in the predictor with 95% confidence intervals; *Z-value*, a test statistic that represents the regression coefficient divided by its standard error, indicating how many standard deviations the coefficient estimate is from zero. And Z-value with *p*-value less than 0.05 was considered significant. *Q_M_*, the Q statistic tests for subgroup differences based on random effects model, and Q_M_-value with *p*-value less than 0.05 was considered significant; ^*^: *p*-value < 0.05.

**Supplementary Table 7. The Egger test for publication bias**

|  | Study size | I^2^ | Bias estimate (SE) | t | *p-*value |
| --- | --- | --- | --- | --- | --- |
| Glx-ACC | 21 | 61% | -3.7459(1.5654) | -2.39 | 0.0272* |
| Glx-PFC | 17 | 62% | -2.6735(1.7270) | -1.55 | 0.1425 |

Notes: *ACC*, anterior cingulate cortex; *PFC*, prefrontal cortex; *Glx*, glutamine + glutamate; *GABA*, γ-aminobutyric acid; *SE,* standard error; ^*^: *p*-value < 0.05.

**Supplementary Table 8. Meta-analysis pooled effect size before and after outliers’ removal for comparison MDD patients at baseline and intervention**

| Random effect model | Results for random effects model analysis | | Test of heterogeneity | | Removed studies |
| --- | --- | --- | --- | --- | --- |
|  |  | |  | |  |
|  |  | SMD (95%CI) | I^2^ | *p-*value for Q test |  |
| **GABA** |  |  |  |  |  |
| PFC | Main analysis | -0.23 (-0.73; 0.28) | 50.2% | 0.0906 | - |
|  | Infl. Cases Removed | - | - | - | No outliers detected |

Notes: *SMD*, Standardized mean difference; *CI*, Confidence intervals; *ACC*, anterior cingulate cortex; *PFC*, prefrontal cortex; *OCC*, occipital cortex; *Glu*, glutamate; *Glx*, glutamine + glutamate; *GABA*, γ-aminobutyric acid; *I^2^*, an index of heterogeneity; *Q*, the Cochran's Q statistic tests for heterogeneity; ^*^: *p*-value < 0.05.

| Random effect model | Test for coefficient | | | | Test for model | | |
| --- | --- | --- | --- | --- | --- | --- | --- |
|  | Predictor | Coefficient (95%CI) | Z-value | *p*-value | R^2^ | Q_M_-val | *p*-value |
| **Glx** |  |  |  |  |  |  |  |
| ACC | Patients’ age | 0.004 (-0.03, 0.04) | 0.25 | 0.80 | 0.00% | 0.06 | 0.80 |
|  | Patients’ gender  (female ratio) | 0.013 (-0.02, 0.04) | 0.87 | 0.38 | 0.00% | 0.76 | 0.38 |
|  | Patients’ sample size | -0.01 (-0.03, 0.01) | -0.71 | 0.48 | 0.00% | 0.51 | 0.48 |
| PFC | Patients’ age | -0.01 (-0.04, 0.01) | -1.07 | 0.28 | 0.00% | 1.15 | 0.28 |
|  | Patients’ gender  (female ratio) | 0.01 (-0.05, 0.06) | 0.28 | 0.78 | 0.00% | 0.08 | 0.78 |
|  | Patients’ sample size | 0.04 (-0.02, 0.10) | 1.17 | 0.24 | 0.00% | 1.37 | 0.24 |
| **Glu** |  |  |  |  |  |  |  |
| ACC | Patients’ age | 0.008 (-0.01; 0.03) | 0.76 | 0.45 | 0.00% | 0.58 | 0.45 |
|  | Patients’ gender  (female ratio) | -0.01 (-0.06; 0.03) | -0.67 | 0.50 | 0.00% | 0.45 | 0.50 |
|  | Patients’ sample size | -0.03 (-0.11; 0.06) | -0.67 | 0.50 | 0.00% | 0.45 | 0.50 |
| PFC | Patients’ age | -0.002 (-0.04, 0.03) | -0.12 | 0.90 | 0.00% | 0.01 | 0.90 |
|  | Patients’ gender  (female ratio) | -0.01 (-0.03, 0.004) | -1.41 | 0.16 | 99.99% | 2.00 | 0.16 |
|  | Patients’ sample size | -0.04 (-0.11, 0.04) | -0.99 | 0.32 | 46.70% | 0.98 | 0.32 |
| **GABA** |  |  |  |  |  |  |  |
| ACC | Patients’ age | 0.006 (-0.02; 0.03) | 0.44 | 0.66 | 0.00% | 0.19 | 0.66 |
|  | Patients’ gender  (female ratio) | 0.02 (-0.02, 0.06) | 0.88 | 0.38 | 19.72% | 0.78 | 0.38 |
|  | Patients’  sample size | -0.01 (-0.04, 0.01) | -0.94 | 0.34 | 0.00% | 0.89 | 0.34 |
| PFC | Patients’ age | -0.02 (-0.19, 0.16) | -0.18 | 0.86 | 0.00% | 0.03 | 0.85 |
|  | Patients’ gender  (female ratio) | 0.04 (-0.06, 0.13) | 0.78 | 0.43 | 0.00% | 0.61 | 0.43 |
|  | Patients’ sample size | 0.08 (-0.03, 0.19) | 1.47 | 0.14 | 57.41% | 2.15 | 0.14 |

**Supplementary Table 9. Results of meta-regression for MDD patients at baseline and intervention**

Notes: *ACC*, anterior cingulate cortex; *PFC*, prefrontal cortex; *OCC*, occipital cortex; *Glu*, glutamate; *Glx*, glutamine + glutamate; *GABA*, γ-aminobutyric acid; *Z-value*, a test statistic that represents the regression coefficient divided by its standard error, indicating how many standard deviations the coefficient estimate is from zero. And Z-value with *p*-value less than 0.05 was considered significant. *Q_M_*, the Q statistic tests for subgroup differences based on random effects model, and Q_M_-value with *p*-value less than 0.05 was considered significant; ^*^: *p*-value < 0.05.

**
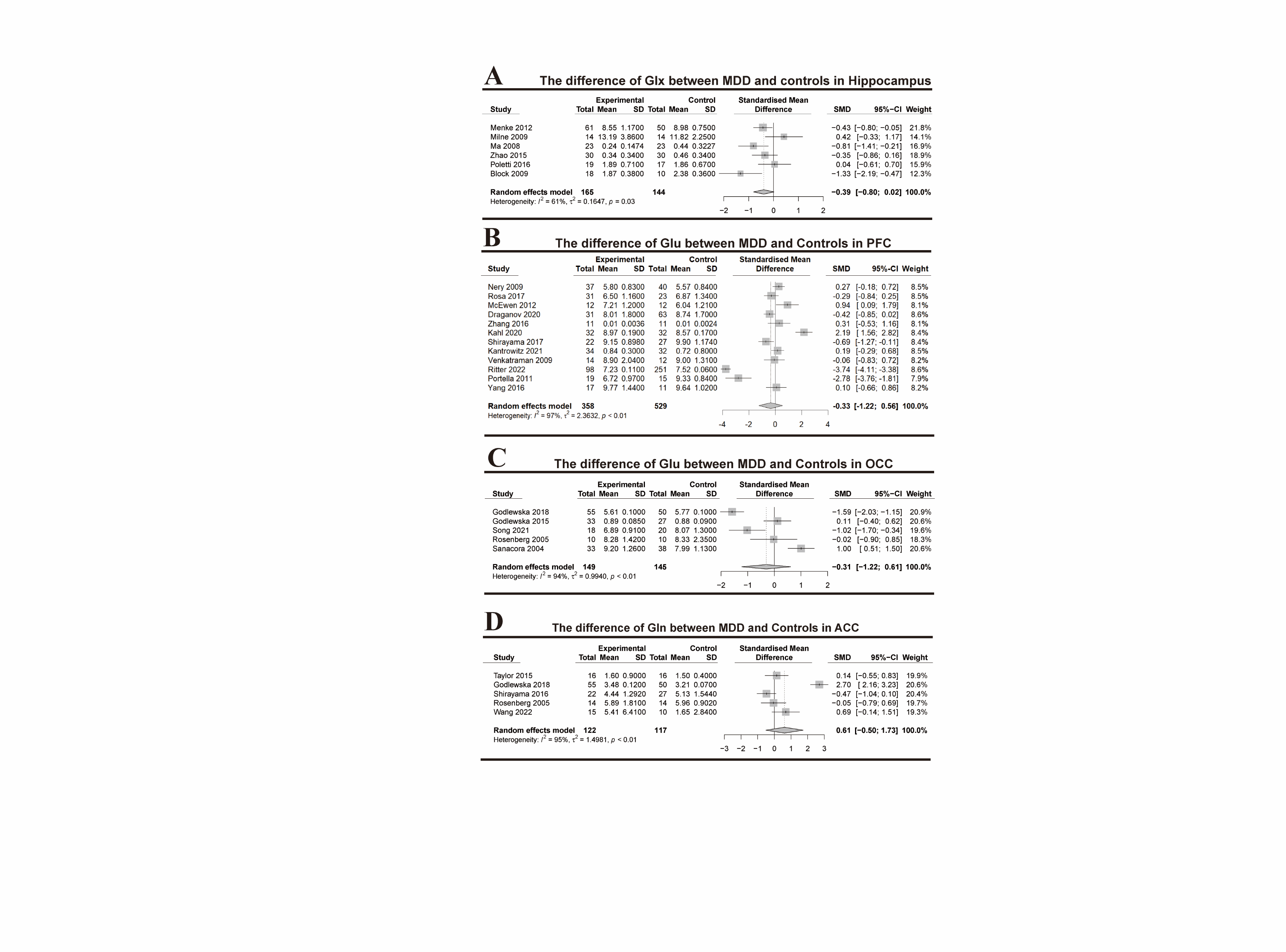
**

**
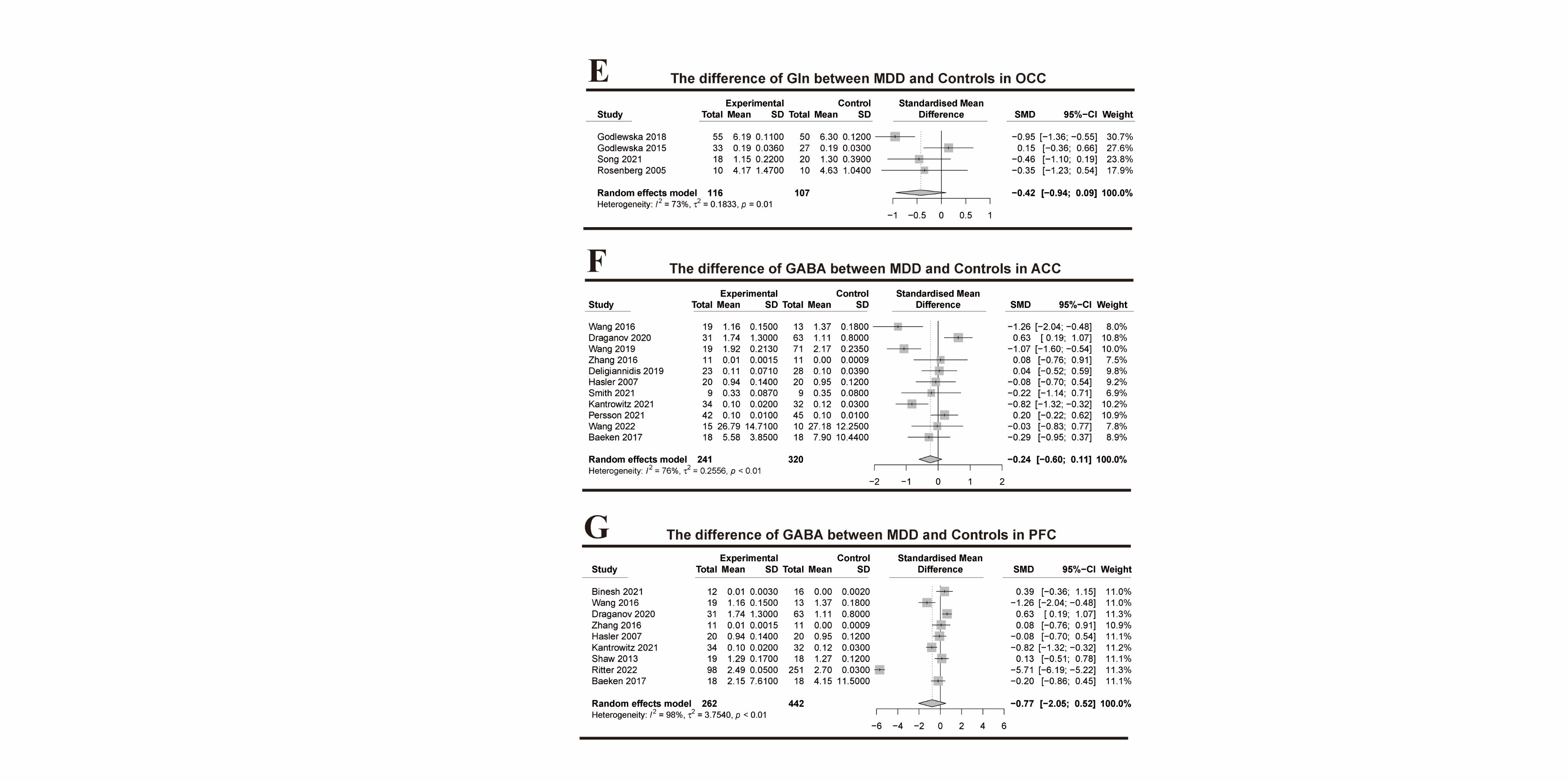
Supplementary Figure 1. Effect sizes of neurometabolites in brain regions that did not represent significant differences between MDD and healthy controls.** Forest plots show the pooled effect sizes (i.e., standardized mean difference with 95% confidence interval) of random-effects meta-analysis on differences in various brain neurometabolite (A, Glx in hippocampus; B, Glu in PFC; C, Glu in OCC; D, Gln in ACC; E, Gln in OCC; F, GABA in ACC; G, GABA in PFC) concentrations between MDD patients and healthy controls. The diamond markers display the pooled effect size of our random effects model. Rows of the forest plot list studies included in the meta-analysis. For each study, the forest plots lay out its author’s name, publication year, study size, effect size and study weight.

Notes: *SMD*, Standardized mean difference; *CI*, Confidence intervals; *ACC*, anterior cingulate cortex; *PFC*, prefrontal cortex; *OCC*, occipital cortex; *Glu*, glutamate; *Gln*, glutamine; *Glx*, glutamine + glutamate; *GABA*, γ-aminobutyric acid; *I^2^*, an index of heterogeneity; *p*, for describing the significance of heterogeneity.


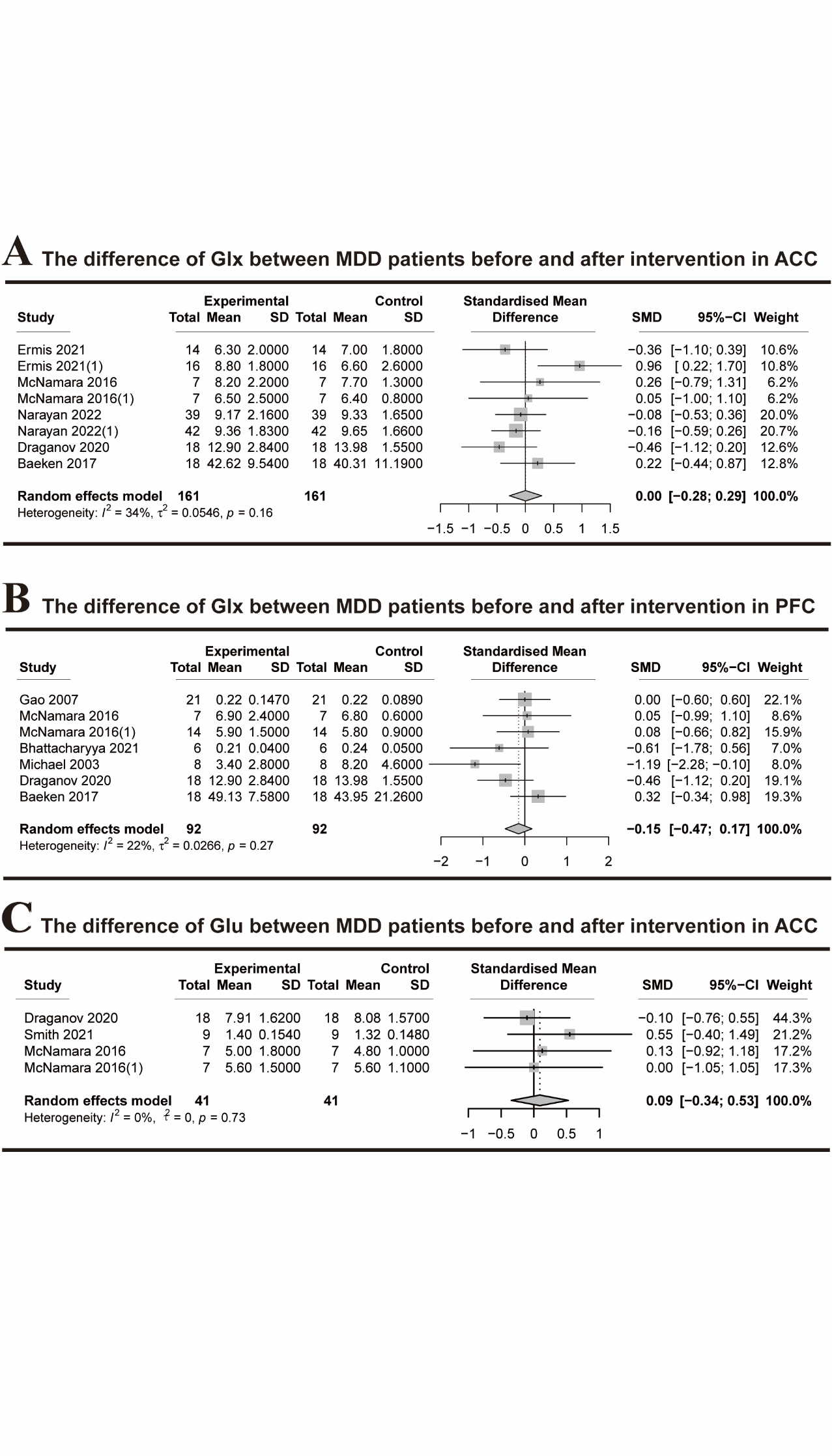


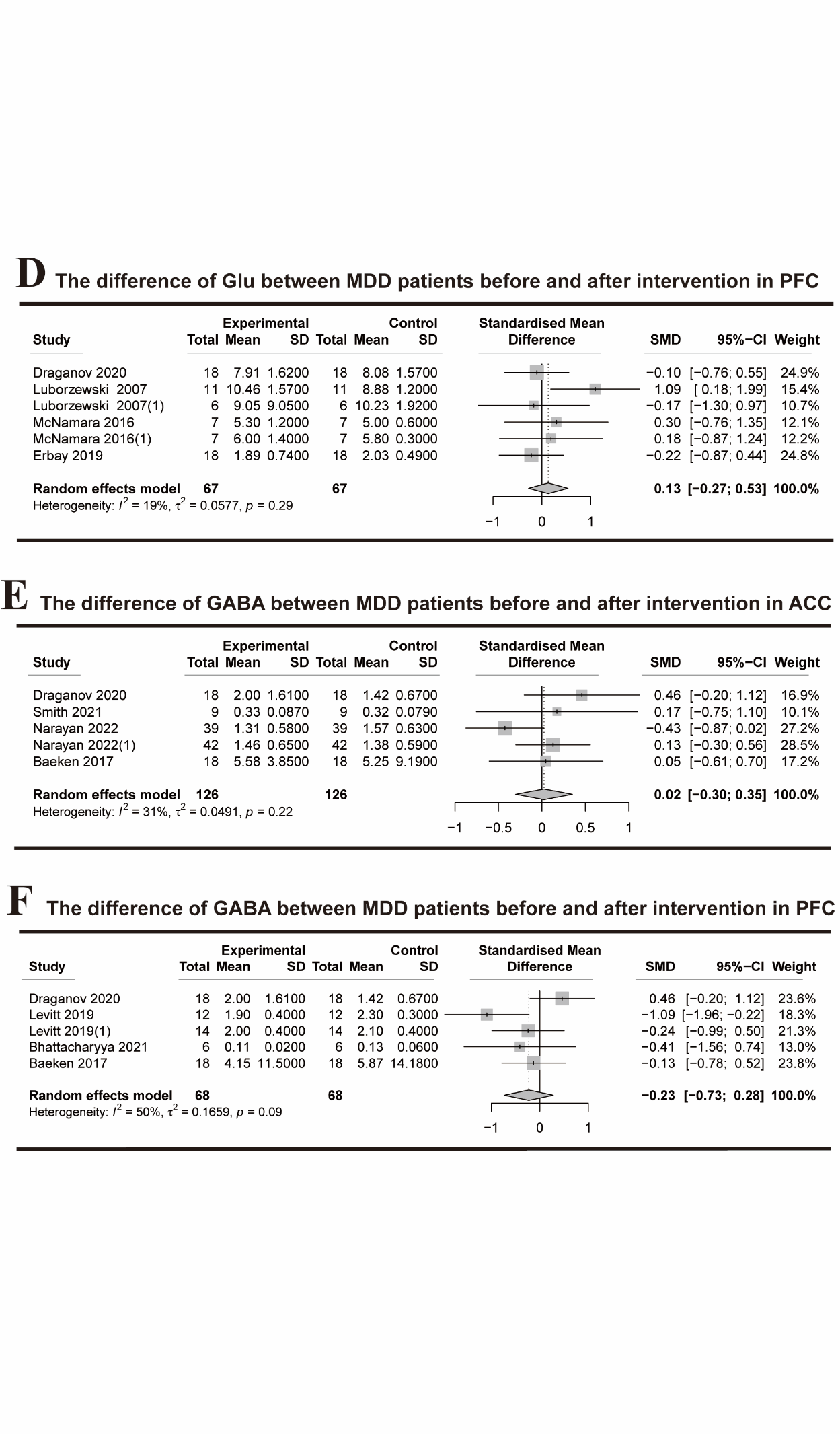


**Supplementary Figure 2. Effect sizes of neurometabolites in brain regions between patients pre- and post-intervention.** Forest plots show the pooled effect sizes (i.e., standardized mean difference with 95%CI) of random-effects meta-analysis on differences in various brain neurometabolite (**A**, Glx in ACC; **B**, Glu in ACC; **C**, Glx in PFC; **D**, GABA in OCC) levels between MDD patients and controls. The diamond markers display the pooled effect size of our random effects model. Rows of the forest plot list studies included in the meta-analysis. For each study, the forest plots lay out its author’s name, publication year, study size, effect size and study weight.

Notes: *SMD*, Standardized mean difference; *CI*, Confidence intervals; *ACC*, anterior cingulate cortex; *PFC*, prefrontal cortex; *OCC*, occipital cortex; *Glu*, glutamate; *Glx*, glutamine + glutamate; *GABA*, γ-aminobutyric acid; *I^2^*, an index of heterogeneity; *p*, for describing the significance of heterogeneity.

**
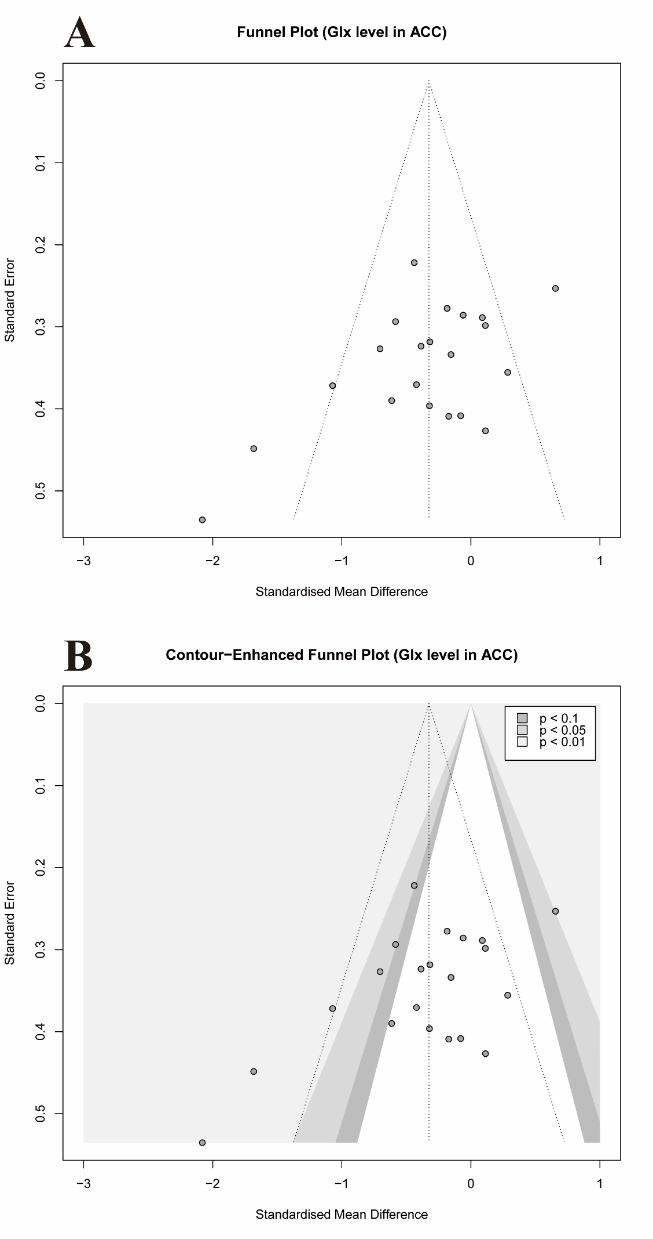

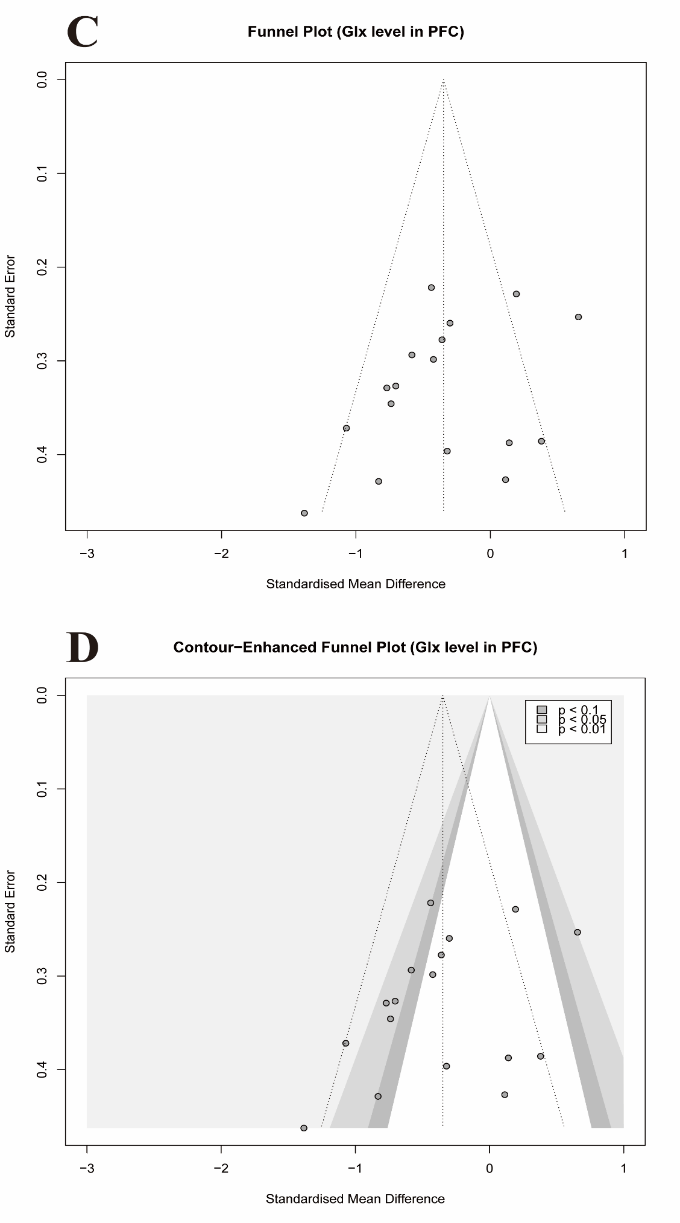
**

**Supplementary Figure 3. Funnel plots for** **comparison between patients and controls with study size above ten and I^2^ less than 75%. A**, Glx in ACC; **C**, Glx in PFC; and contour-enhanced (CE) funnel plots; **B**, Glx in ACC; **D**, Glx in PFC show the effect size of each study (expressed as the standardized mean difference) on the x-axis, and the standard error (from large to small) on the y-axis. The vertical line in the middle of the funnel shows the average effect size. CE funnels use colors to signify the significance level of each study in the plot.

Notes: *CE funnel plot,* contour-enhanced funnel plot; *ACC*, anterior cingulate cortex; *PFC*, prefrontal cortex.


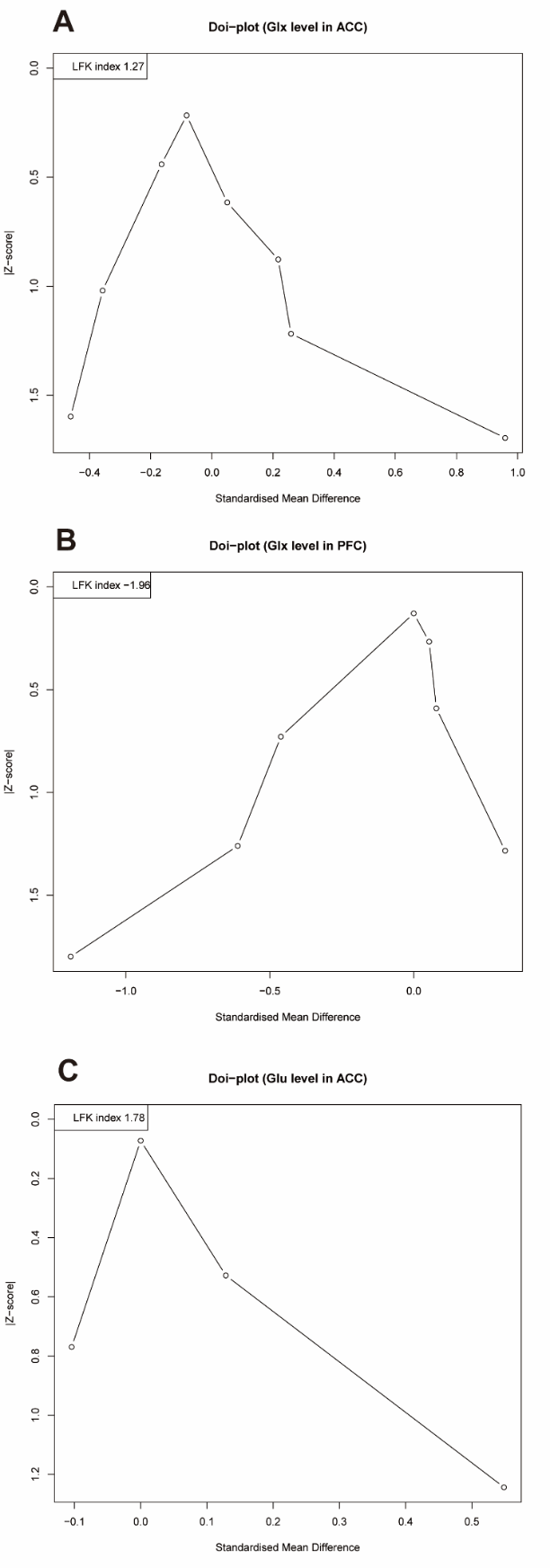

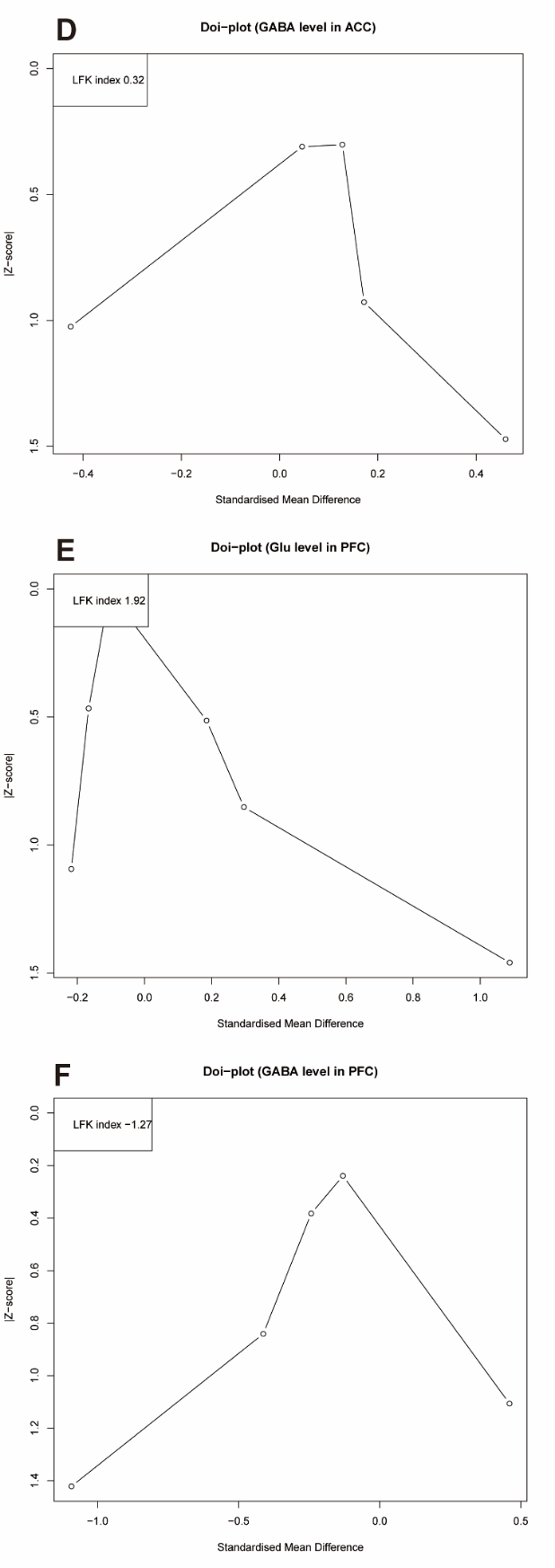


**Supplementary Figure 4. Doi plot analysis and LFK index of publication bias for comparison in patients pre- and post-intervention below ten and I^2^ less than 75%**. **A**, Glx in ACC; **B**, Glx in PFC; C. Glu in ACC; **D**, Glu in PFC; **E**, GABA in ACC; **F**, GABA in PFC. The Doi plot for publication bias showed no asymmetry and no evidence of bias by the asymmetry index for AGABA in ACC (LFK < 1).

Notes: *LFK*, Luis Furuya-Kanamori index.

**
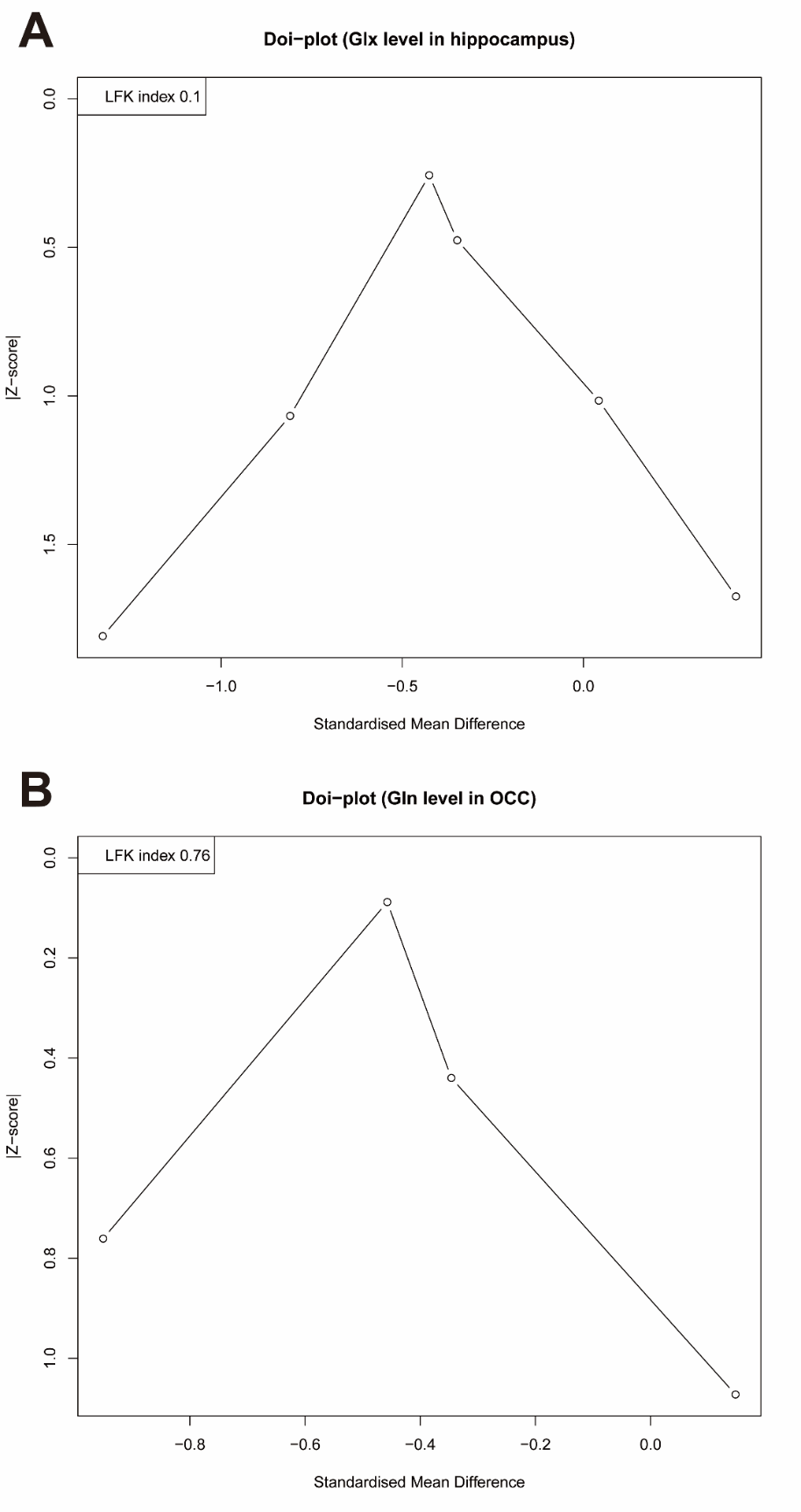
**

**Supplementary Figure 5. Doi plot analysis and LFK index of publication bias for comparison between patients and controls with study size below ten and I^2^ less than 75%.** **A**, Glx in hippocampus; **B**, Gln in OCC. The Doi plot for publication bias showed no asymmetry and no evidence of bias by the asymmetry index (LFK < 1).

Notes: *LFK*, Luis Furuya-Kanamori index.
